# Investigation of the transient heat transfer to a supersonic air jet impinging on a high-temperature plate based on a discrimination-experiment method

**DOI:** 10.1371/journal.pone.0264968

**Published:** 2022-03-14

**Authors:** Ming-Xin Gao, Jian Yang, Yue Zhang, Hua Song

**Affiliations:** 1 School of Materials and Metallurgy, University of Science and Technology Liaoning, Anshan, China; 2 School of Mechanical Engineering and Automation, University of Science and Technology Liaoning, Anshan, China; Tongji University, CHINA

## Abstract

A discrimination-experiment method is developed to investigate the transient heat transfer of air jet impingement by discretizing the solid domain into mutually adiabatic test cylinders. This method can not only reduce the influence of the transverse heat transfer of a solid domain on the heat transfer characteristics of the jet but can also simplify the two-dimensional or three-dimensional heat conduction problem into a one-dimensional problem. Moreover, the discrimination-experiment method eliminates the embedment of thermocouples into the solid domains, further improving the accuracy and reliability of the proposed method. The transient heat transfer characteristics of a supersonic air jet impinging on a high-temperature target (860°C) and the effects of thermo physical parameters, such as the density, specific heat capacity, thermal conductivity and nozzle-to-target distance (*H/D* = 3, 4, and 5) are analyzed in detail using the discrimination-experiment method. The results provide important guidance for the thermal design of supersonic air jet impingement.

## Introduction

Jet impingement is an extremely effective method to enhance the heat transfer by spraying the desired fluid on the target surface. With compressed air as a medium, the air jet impingement has been widely used in a wide range of heat transfer applications, such as the cooling of electronic components, surface strengthening treatments of steel or glass, and forced cooling of power plants, due to its environmentally friendly nature, high heat transfer efficiency, and excellent cooling uniformity. However, compared with other jet media, such as water or water mist, the air jet exhibits a poor heat transfer capacity, which seriously limits its application. The convective heat transfer coefficient of a conventional air jet can only reach about 350–450 W·m^-2^·°C^-1^ [1, 2]. The utilization of a pulse air jet can improve the heat transfer capacity by 85%, but it still cannot meet the practical requirements [3, 4]. Another way to enhance the heat transfer capacity of air jets is to increase the jet velocity. With Laval nozzles as the core component, the supersonic air jet impingement has received extensive attention in several heat transfer applications. For instance, Lee et al. [5] observed that the maximum heat transfer capacity of a supersonic air jet is about a factor of 10 higher than that of conventional air jets. Parker et al. [6] and Kim et al. [7] found that the convective heat transfer coefficients could reach 2000–10000 W·m^-2^·°C^-1^ when supersonic air was utilized as the jet medium.

The research on the heat transfer characteristics of air jet impingement, especially the convective heat transfer coefficient, is critical for predicting the heat transfer rate and formulating the process parameters in thermal engineering fields. However, most of the existing studies focused on the steady-state method [8], in which a stable state is maintained by continuously supplying a known constant energy to the heat transfer target during the jet impingement process. Then, the convective heat transfer coefficient is calculated as *h* = *q* / (*t*_*s*_ − *t*_∞_), where *h* refers to the convective heat transfer coefficient, *q* denotes the heat flux (i.e., the supplied energy), *t*_*s*_ represents the temperature of the heat transfer surface, and *t*_∞_ corresponds to the jet temperature. However, the steady-state method cannot be utilized to conduct different processes, such as surface quenching or continuous casting cooling, because the heat transfer characteristics vary with time during the impingement process [2, 9, 10].

On the other hand, the transient method takes into account the variation of the heat transfer characteristics with time, mainly relying on numerical simulations and experimental investigations. The numerical simulation method is widely used because of its cost-effectiveness and high stability [11–15]. However, it is difficult to obtain a mathematical model of supersonic air-quenching considering the efficiency and accuracy of numerical simulations [6, 16]. On the one hand, a large pressure gradient, large temperature gradient, and large velocity gradient exist simultaneously [17, 18]. On the other hand, the thermophysical parameters, such as the density, specific heat, and thermal conductivity, of both the jet and the heat transfer target are significantly altered with temperature. Compared with the transient numerical simulations, the experimental investigations offer higher accuracy and reliability. Furthermore, the inverse method is the main approach to obtain the convective heat transfer coefficient by measuring the temperature gradient of the impingement target and solving the heat conduction differential equation when conducting experimental investigations [19]. However, the disadvantage of the inverse method is that it requires the installation of thermocouples inside the solid domains to obtain the internal temperature. For instance, Fletcher et al. installed five thermocouples inside the test plate to study the heat transfer of the quenching process [20]. Three thermocouples were embedded into a hollow sphere at a depth of 2.6 mm from the outer surface to study the heat transfer of the hollow sphere quenching [21].

Similarly, five thermocouples were embedded into a cylinder to assess the validity of the determination method of the heat transfer coefficient by Sugianto et al. [22]. Moreover, 10 test points were considered to study the water jet impinging on a high-temperature (900°C) stainless-steel plate by Dou et al. [23], and three thermocouples were installed at each test point to detect the temperature of the top, inner, and lower surfaces. Furthermore, 15 thermocouples were installed in the solid domain at a distance of 2 mm under the impingement surface to study the conventional air jet impinging on a high-temperature (800°C) target by Zhou et al. [2]. Clearly, the installation of thermocouples through the mounting of holes leads to inevitable errors in the temperature measurement position [1] and affects the temperature distribution in solid domains. Therefore, it is of utmost importance to reduce the number of thermocouples to improve the accuracy of the experiments.

**Fig 1:**
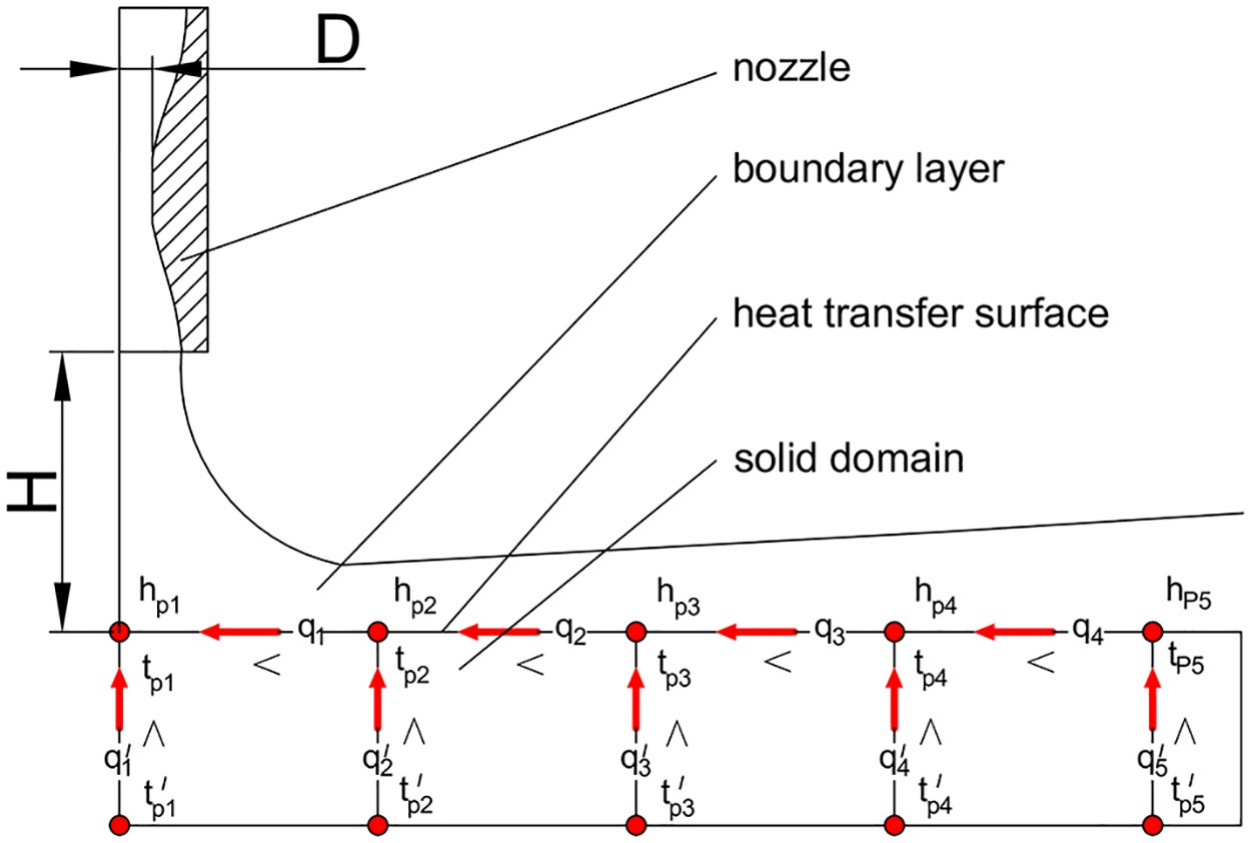
Heat transfer network analysis of the conjugate heat transfer process.

Moreover, as shown in Fig 1, the heat transfer of the jet impingement is a conjugate process [7, 24, 25], and the heat transfer characteristics are strongly influenced by the type and size of the solid domains [11, 26, 27]. Hence, the heat transfer intensity at different positions is different, leading to a temperature difference on the heat transfer surface and a transverse heat conduction from the higher temperature region to the lower temperature region. Additionally, the transverse heat transfer in the solid domains affects the surface temperature distribution [27]. The average heat transfer of the conventional air jet impingement is altered by 9% when the solid material consists of copper, steel, or a nickel-based alloy [24]. Besides, the difference will increase with the increase in the convective heat transfer intensity.

Therefore, the current study aims to develop a discrimination-experimental method for the heat transfer of the transient air jet impingement. This method is not only suitable for large temperature differences and severe heat transfer processes but can also improve the accuracy by reducing the influence of the transverse heat transfer and reducing the number of thermocouples in the solid domains. Moreover, the transient heat transfer characteristics of the supersonic air jet impingement on high-temperature targets and the effects of thermophysical parameters, such as the density, specific heat capacity, and thermal conductivity, as well as the nozzle-to-target distance (*H/D* = 3, 4, and 5) are systematically analyzed.

## Discrimination-experiment method

### Principle and structure of the discrimination-experiment method

**Fig 2:**
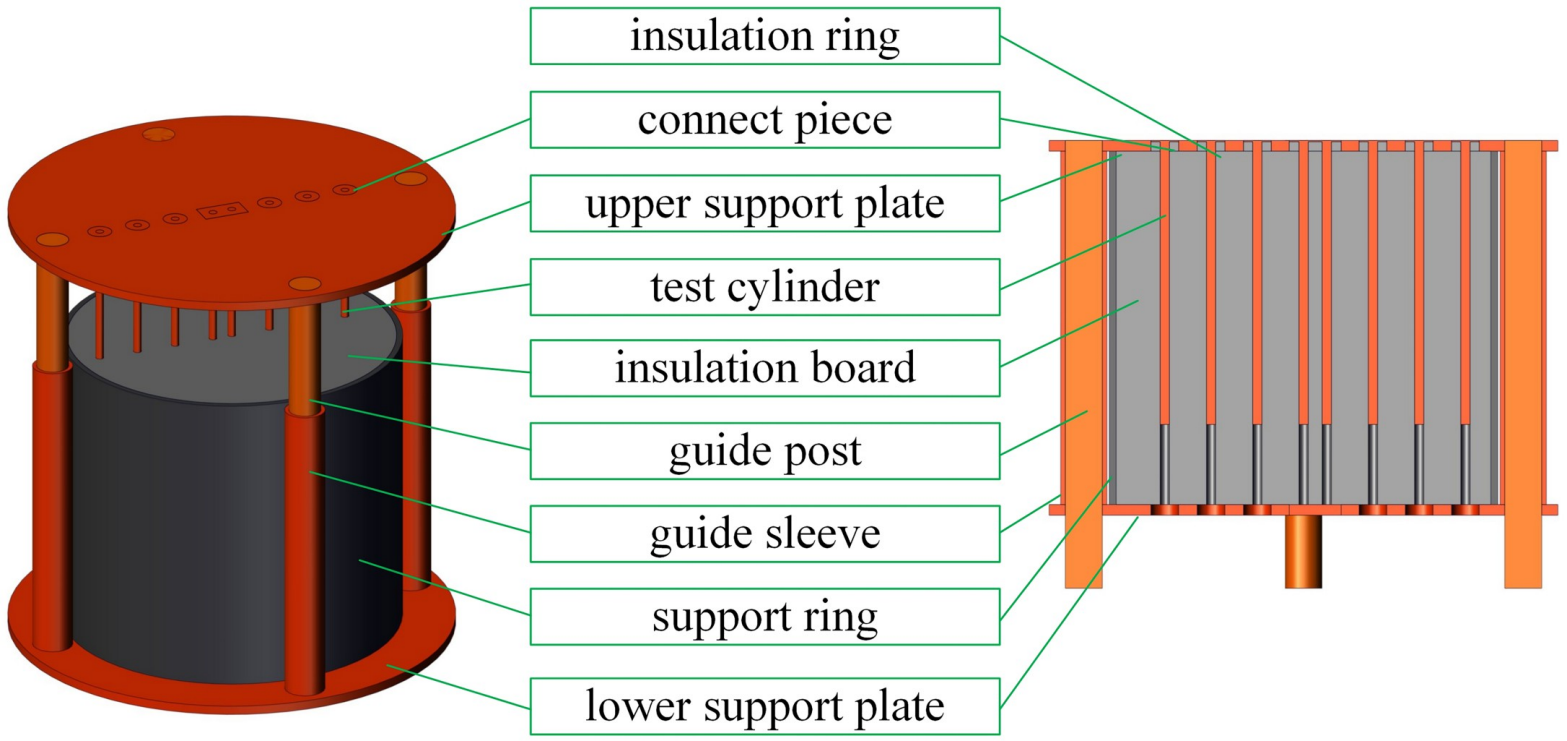
Detailed view of the device of the discrimination-experiment method.

The principle of the discrimination-experiment method is based on dividing the solid domains into independent test points at different positions by setting test cylinders, which are perpendicular to the heat transfer interface. Additionally, the transverse heat transfer between each test cylinder is separated by an insulating material. Considering the circular jet impingement on the plate as an example, the detailed view of the device of the discrimination-experiment method is shown in Fig 2. The material of the upper support plate and the connect piece is the same as that of the test cylinder. Round or square holes are machined at the test positions on the upper support plate. The size of the connect piece is the same as that of the hole on the upper support plate. The center of the connect piece is a hole with the same diameter as that of the test cylinder.

The outer and inner surfaces of the connect piece are welded with the upper support plate and test cylinder, respectively. After welding, the upper support plate, test cylinders, and connect piece are machined to the same roughness to avoid the influence on the flow field. The thickness of the connect piece should be as small as possible to minimize its influence on the temperature distribution. The insulation board should be a machinable material, such as a high-temperature resistant glass fiber or an aluminum silicate ceramic. The holes are slightly larger than the test cylinder and are machined on the insulation board at each test position. Hence, the test cylinder can slide smoothly into the hole. The thickness of the insulation board should be higher than the length of the test cylinder to block the heat transfer from the lower surface of the test cylinder to the environment. The support ring and the lower support plate are arranged on the outer and lower sides of the insulation board to strengthen the device. The guide columns and guide sleeves are fixed on the lower support plate and upper support plate, respectively. A high-temperature resistant grease is applied between the guide column and the guide sleeve to ensure that the guide column slides smoothly in the guide sleeve.

The steps in the discrimination-experiment method are as follows:

1. Before heating, the upper support plate is moved upward with the test cylinder to ensure that all parts of the test cylinder are in direct contact with the heating environment, and a block is placed between the upper support plate and the support sleeve.
2. The device is heated to the desired temperature in a furnace.
3. Before removing the device from the furnace, the block is removed, the upper support plate is returned with the test cylinder, and the top surface is covered with an insulating material.
4. The device is taken out from the furnace with the insulation cover.
5. The insulation cover is removed, and the jet is turned on at the same time.
6. The upper surface temperature of the device is recorded as the boundary condition for numerical computations.

### Numerical computations of the discrimination-experiment method

**Fig 3:**
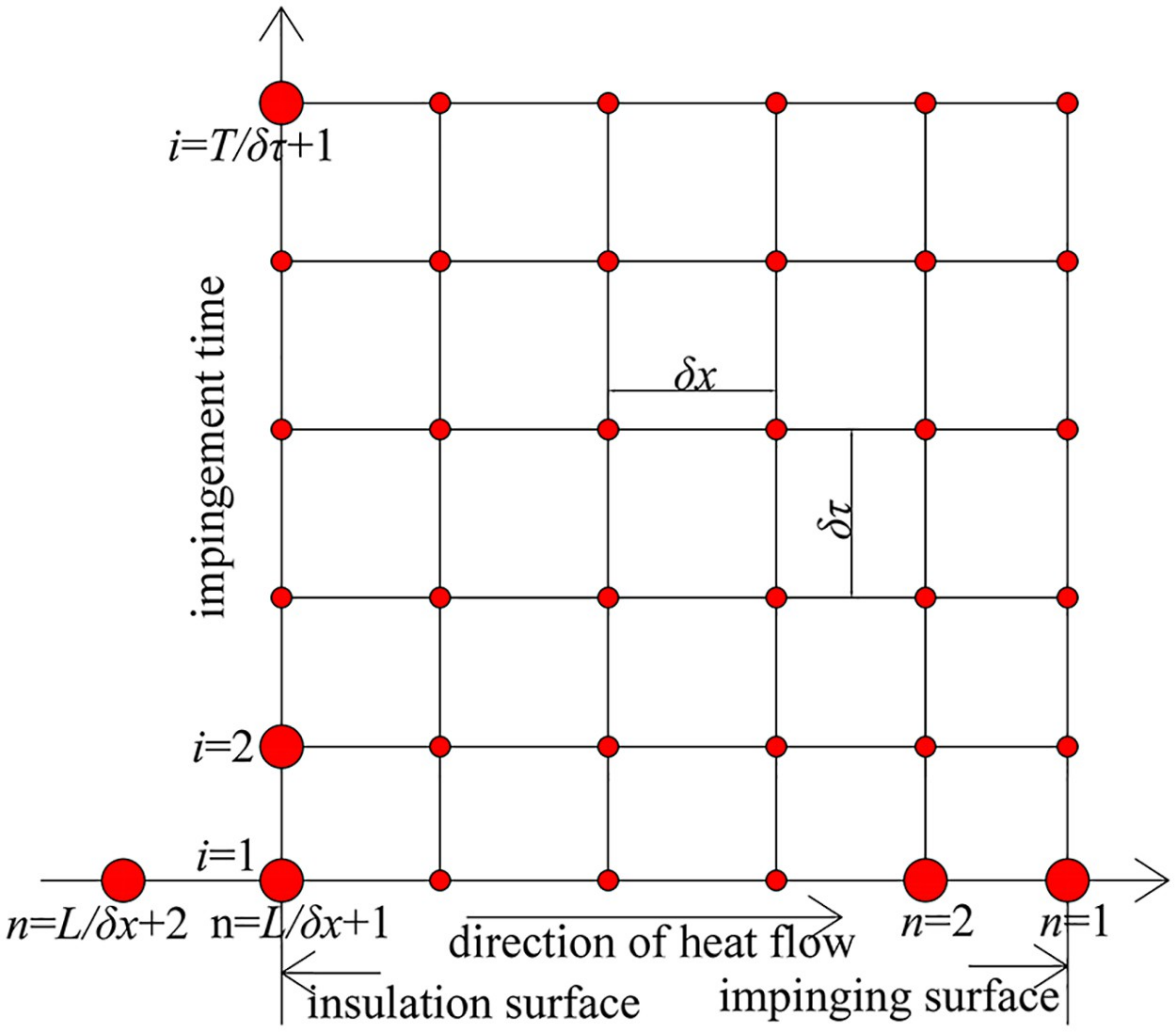
Numerical computation nodes of the discrimination-experiment method.

The side and bottom of the test cylinders are insulated. Hence, heat transfer is only allowed between the upper surface and the jet. Assuming that there is no temperature gradient in the same cross section of the test cylinder, each test cylinder satisfies the one-dimensional heat conduction condition. The numerical computation nodes used in the discrimination-experiment method are shown in Fig 3, where the abscissa and ordinate represent the axial position of each test cylinder and jet impingement time, respectively; *δτ* and *δx* denote the corresponding time step and space step. Moreover, *L* and *T* correspond to the length of the test cylinder and the end time of the impingement, respectively; *n* refers to the location number of the node; *n* = 1 and *n* = *L* / *δx* + 1 correspond to the nodes of the upper and lower surfaces of the test cylinder, respectively; *n* = *L* / *δx* + 2 refers to an imaginary point, and its temperature is same as *n* = *l* / *δx*; *i* represents the time number of the node, where *i* = 1 and *i* = *T* / *δτ* + 1 are the nodes of the start and end times of the impingement, respectively.

The heat conduction differential equation of each test cylinder is written as:

$$\rho c_{p}\frac{\partial t}{\partial \tau }=\frac{\partial }{\partial x}\left(\lambda \frac{\partial t}{\partial x}\right)$$ (1)

where *ρ*, *cp*, and *λ* represent the density, specific heat capacity, and thermal conductivity of the test cylinder, respectively. By discretizing Eq (1) using the finite-difference method, the temperature distribution of each test cylinder at a given time can be written as:

$$\rho c_{p}\frac{t_{n}^{i+1}-t_{n}^{i}}{\delta \tau }=\lambda \frac{t_{n-1}^{i}-2t_{n}^{i}+t_{n+1}^{i}}{{\left(\delta x\right)}^{2}}\;n=2,\ldots ,\frac{L}{\delta x}+1\;\text{and}\;i=1,\ldots ,\frac{T}{\delta \tau }+1$$ (2)

where $t_{n}^{i}$ refers to the temperature of node *n* at time *i*. Furthermore, Eq (2) can be rewritten as:

$$t_{n}^{i+1}=A\left(t_{n-1}^{i}+t_{n+1}^{i}\right)+Bt_{n}^{i}$$ (3)

where

$$A=\frac{\lambda }{\rho c_{p}}\frac{\delta \tau }{{\left(\delta x\right)}^{2}},B=1-\frac{2\lambda }{\rho c_{p}}\frac{\delta \tau }{{\left(\delta x\right)}^{2}}$$ (4)

One should note that the calculations converge only when:

$$B=1-\frac{2\lambda }{\rho c_{p}}\frac{\delta \tau }{{\left(\delta x\right)}^{2}}\geq 0$$ (5)

Eq (5) can be rewritten as:

$$\delta \tau \leq \frac{\rho c_{p}}{2\lambda }{\left(\delta x\right)}^{2}$$ (6)

The initial temperature of the test cylinder can be written as:

$$t_{n}^{1}=t_{0}\;n=1,\ldots ,\frac{L}{\delta x}+1$$ (7)

where *t*_0_ represents the set furnace temperature.

The time-dependent temperature of the upper surface of the test cylinder, measured experimentally, is fitted to the polynomial equation according to:

$$t_{1}^{i}=f\left(\left(i-1\right),\delta \tau \right)=a_{1}{\left(\delta \tau \left(i-1\right)\right)}^{8}+a_{2}{\left(\delta \tau \left(i-1\right)\right)}^{7}+,\ldots ,a_{8}\left(\delta \tau \left(i-1\right)\right)+a_{9}\;i=2,\ldots ,\frac{T}{\delta \tau }+1$$ (8)

where $t_{1}^{i}$ represents the average temperature of the upper surface of the test cylinder at time *i*, and *a*_1_, *a*_2_, … *a*_9_ are the coefficients of the polynomial equation.

The isolation surface condition of the test cylinder can be written as:

$$t_{\frac{L}{\delta x}+2}^{i}=t_{\frac{L}{\delta x}}^{i}\;i=1,\ldots ,\frac{T}{\delta \tau }$$ (9)

According to the energy conversation law, the heat transfer surface condition can be written as:

$$\lambda \frac{t_{2}^{i}-t_{1}^{i}}{\delta x}+h^{i}\left(t_{\infty }-t_{1}^{i}\right)=\rho c_{p}\frac{\delta x}{2}\frac{t_{1}^{i+1}-t_{1}^{i}}{\delta \tau }\;i=1,\ldots ,\frac{T}{\delta \tau }-1$$ (10)

where *t*_∞_ and *t*_*s*_ represent the temperature of the jet and the impingement surface, respectively, and *h*^*i*^ represents the convective heat transfer coefficient of the heat transfer surface at time *i*. Eq (10) can be rewritten as:

$$h^{i}=\rho c_{p}\frac{\delta x(t_{1}^{i}-t_{1}^{i+1})}{2\delta \tau (t_{1}^{i}-t_{\infty })}+\lambda \frac{t_{2}^{i}-t_{1}^{i}}{\delta x(t_{1}^{i}-t_{\infty })}\;i=1,\ldots ,\frac{T}{\delta \tau }-1$$ (11)

In order to ensure the reliability of the experiment, the test cylinder should be long enough to meet the following condition during the discrimination-experiment method:

$$t_{\frac{L}{\delta x}+1}^{i}=t_{0}\;i=1,\ldots ,\frac{T}{\delta \tau }$$ (12)

## Experimental setup of the supersonic air jet impingement on a high-temperature plate

**Fig 4:**
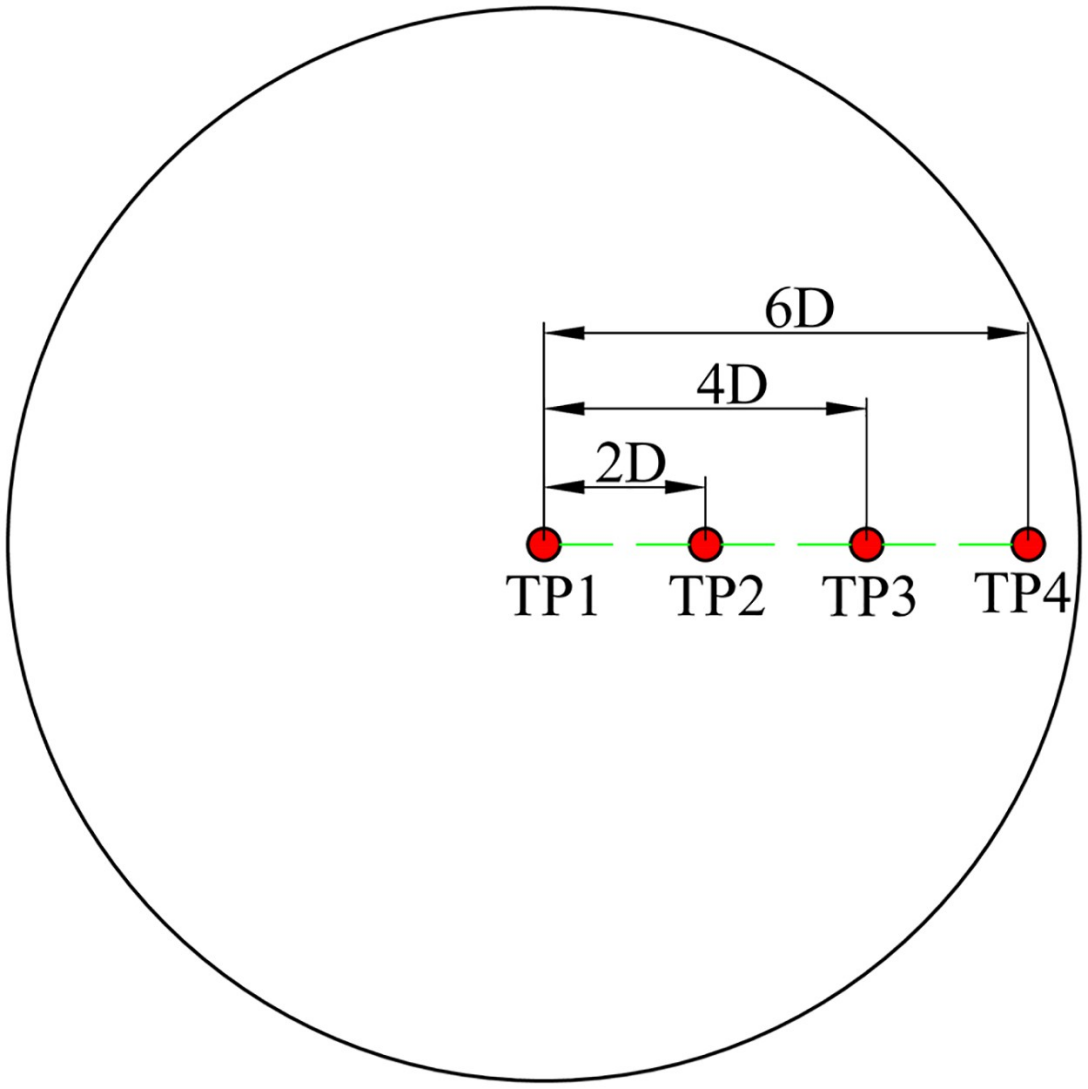
Arrangement of test points in the experiment.

A Laval nozzle with an inlet diameter, throat diameter, and outlet diameter of 0.03, 0.012, and 0.0132 m, respectively, is used in the experiment. The air pressure and temperature of the nozzle inlet are 0.2 MPa and 30°C, respectively. The initial temperature is 860°C. The impingement process lasts for 30 s. The experiment is repeated twice, and the average value is taken. The arrangement of the test points is shown in Fig 4. TP1–TP4 are used to obtain the heat transfer characteristics at different diameters of the interface, where TP1 is located at the jet center, and TP2, TP3, and TP4 are located at *R/D* = 2, *R/D* = 4, and *R/D* = 6, respectively; *R/D* represents the nondimensional distance of the test position. The diameter and length of the test cylinders are 0.004 and 0.10 m, respectively.

### Experimental setup

**Fig 5:**
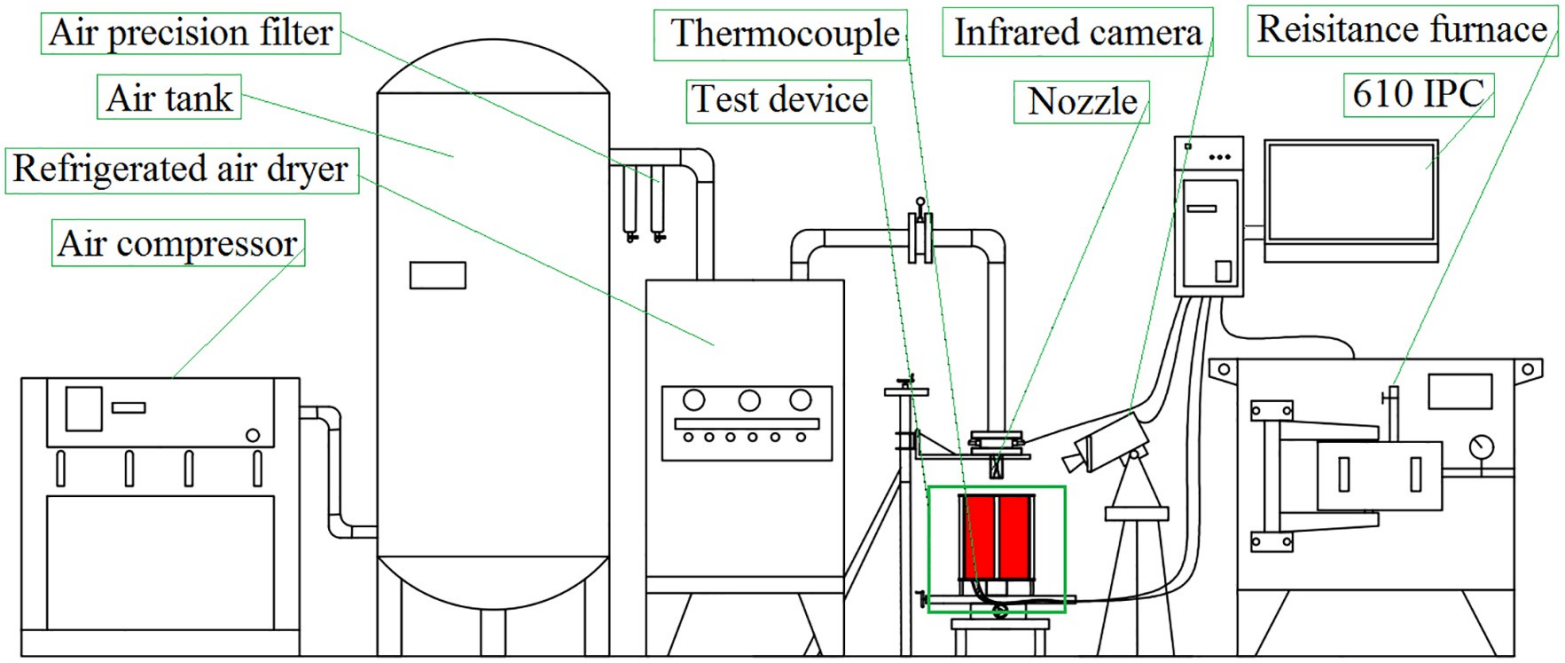
Schematic illustration of the experimental setup.

**Fig 6:**
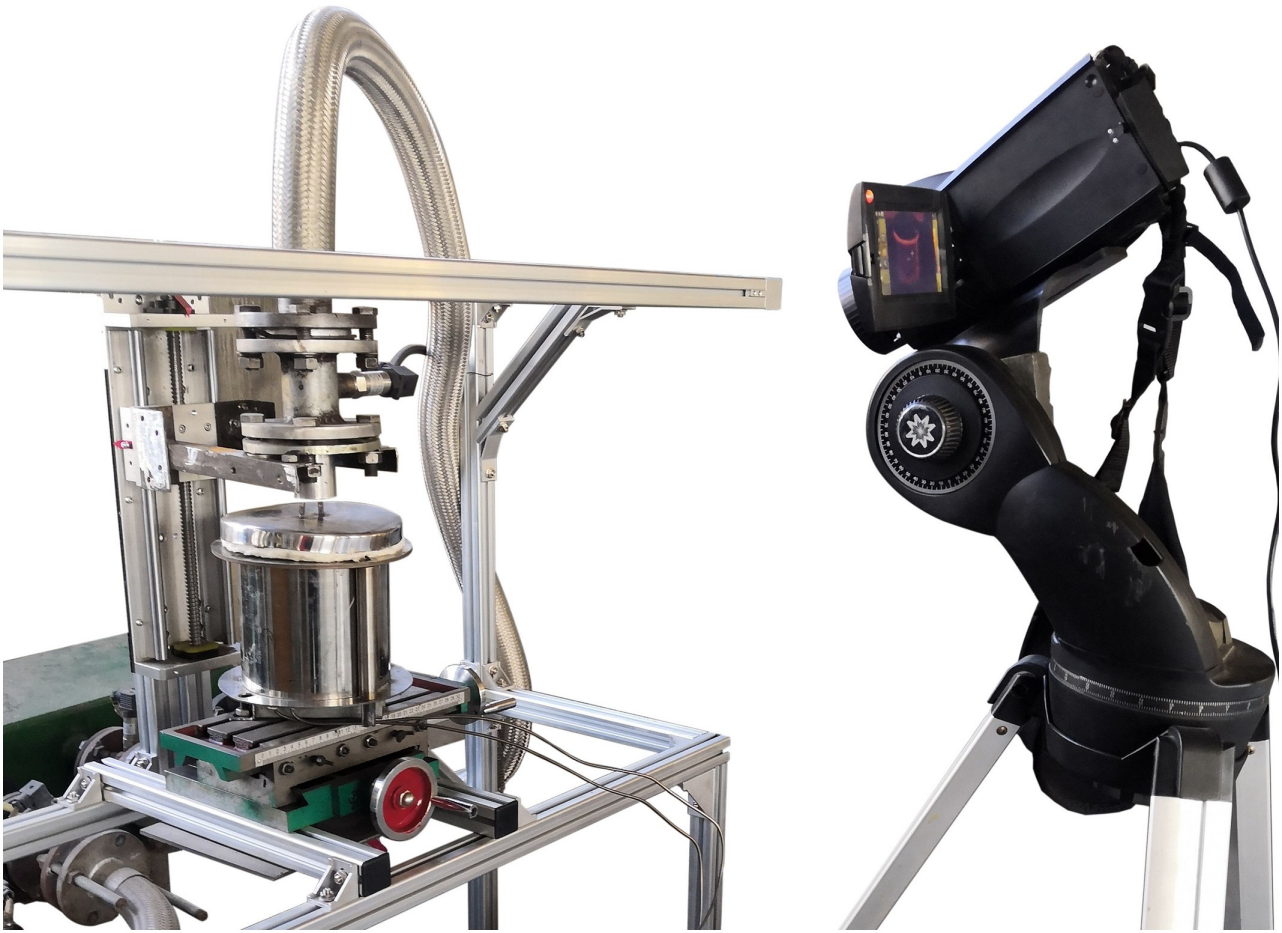
Partial digital photograph of the experimental setup.

The schematic illustration and partial digital photograph of the experimental setup are shown in Figs 5 and 6, respectively. There are four major parts in the experimental apparatus, including the heating unit, jet supply unit, jet impingement unit, and data acquisition and processing unit.

The heating unit is a box-type resistance furnace. The capacity and accuracy of the furnace are 1200°C and ±1°C, respectively. Dry and stable compressed air is provided by the jet supply unit, which includes an LG100 screw air compressor, an HH-2 K-25 air tank, two C-010 compressed air precision filters, and a YD-100HP refrigerated air dryer. The air precision filter and refrigerated air dryer are used to filter the moisture and control jet temperature, respectively. The air tank is used to stabilize the pressure. For the jet impingement unit, the Laval nozzle is connected with the flexible pipe of the air supply unit and fixed on the lifting platform to adjust the nozzle-to-target distance. The data acquisition and processing unit consists of an air pressure sensor and an infrared camera. A pressure sensor is installed in the pipeline between the refrigerated air dryer and the nozzle to measure the airflow pressure through the nozzle. An infrared camera is used to obtain the temperature distribution of the heat transfer surface. The distance and angle between the camera and the target surface are 1 m and 45°, respectively. The infrared camera is a Testo885-2, the thermal sensitivity is less than 30 mK, the spectral range is 7.5–14 μm, and the measurement range is 0–1200°C. The results are collected and analyzed using an IPC-610L industrial computer.

In order to eliminate the influence of the solid-phase transformation, austenitic 304 stainless steel is used as the test cylinder. The chemical composition (in mass %) and thermophysical parameters of the 304 stainless steel are shown in Tables 1 and 2, respectively [28].

**Table 1:** Chemical composition (in mass %) of the austenitic 304 stainless steel.

Composition	C	Cr	Ni	Mo	Mn	Si
mass %	0.04	19	9.3	0	1.0	0.5

**Table 2:** Thermophysical parameters of the austenitic 304 stainless steel.

Temperature (°C)	50	250	350	450	550	650	750	850	950	1050
*ρ* (kg·m^-3^)	7930	7921	7870	7820	7770	7720	7670	7620	7570	7520
*c*_*p*_ (J·kg^-1^·°C^-1^)	450	481	509	529	546	561	576	591	605	619
*λ* (W·m^-1^·°C^-1^)	12.1	14.2	15.9	17.4	19.0	20.5	21.9	23.3	24.7	26.0

By introducing the thermophysical parameters into the polynomial expressions, the following equations are obtained:

$$\lambda =1.823\times 10^{-11}t^{4}-4.774\times 10^{-8}t^{3}+4.137\times 10^{-5}t^{2}+1.506\times 10^{-3}t+11.92$$ (13)

$$c_{p}=4.297\times 10^{-10}t^{4}-1.007\times 10^{-6}t^{3}+7.256\times 10^{-4}t^{2}+1.421\times 10^{-2}t+447.4$$ (14)

$$\rho =5.289\times 10^{-7}\;t^{3}-1.074\times 10^{-3}\;t^{2}+0.159t+17928$$ (15)

**Fig 7:**
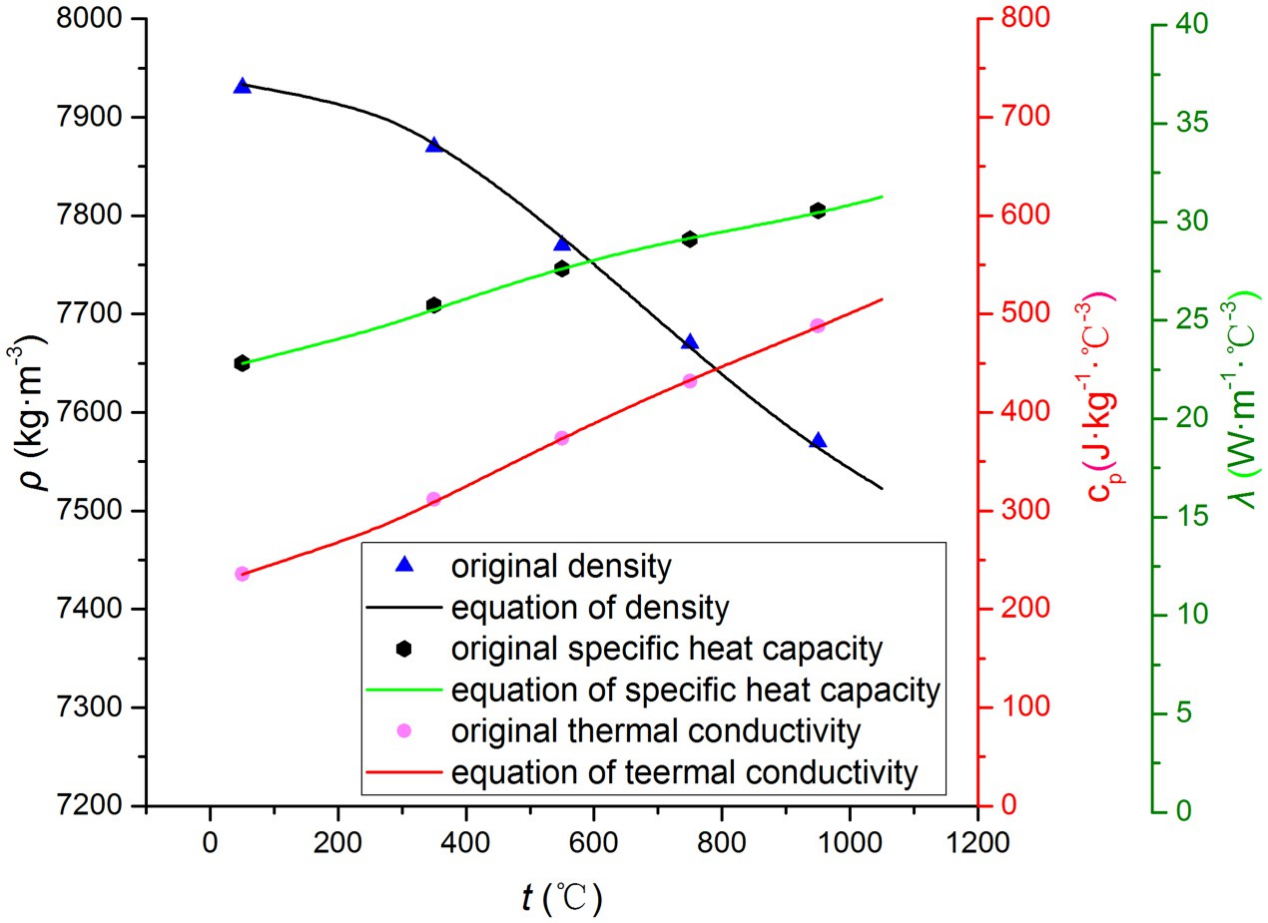
Comparison between the fitting polynomials and the original parameters.

The original parameters and their fitting polynomial curves are shown in Fig 7.

The principle of an infrared camera is based on the measured radiation power of the observed surface. Hence, it is critical to obtain the emissivity of materials under experimental conditions. The empirical values of the emissivity are mostly used in the experiment of the jet impingement heat transfer [29, 30]. However, the emissivity of a material is not constant and depends on the environmental temperature, surface characteristics, shooting distance, and humidity [31–33]. Another common approach is to coat the test surface with a black paint of known emissivity [10, 24], but it is not suitable for the study of the jet impingement heat transfer because it affects the flow field and temperature field at the interface, altering the accuracy of the measured results.

Herein, the contrast method is employed to obtain the emissivity of the heat transfer surface, as detailed below. Firstly, the whole test device is heated to a certain temperature in a calibrated heating furnace. Secondly, the surface is covered with an insulating material to reduce heat consumption. Thirdly, the device is taken out from the furnace and placed in an image collection area. Fourthly, the insulating cover is quickly removed, and the thermal image is immediately recorded. Finally, the emissivity is adjusted until the infrared camera shows the same temperature as the furnace. In order to take into account the influence of the temperature, the test was carried out at 250°C, 450°C, 650°C, and 850°C. The emissivity of the experimental material ranged from 0.37 to 0.45. Herein, 0.41 is taken as the emissivity of the material.

### Time step and space step independence

Based on the parameters listed in Table 2, the maximum of (*ρc*_*p*_) / *λ* is 2.68 × 10^5^. Therefore, according to Eq (6):

$$\delta t\leq 1.34\times 10^{5}{\left(\delta x\right)}^{2}$$ (16)

**Fig 8:**
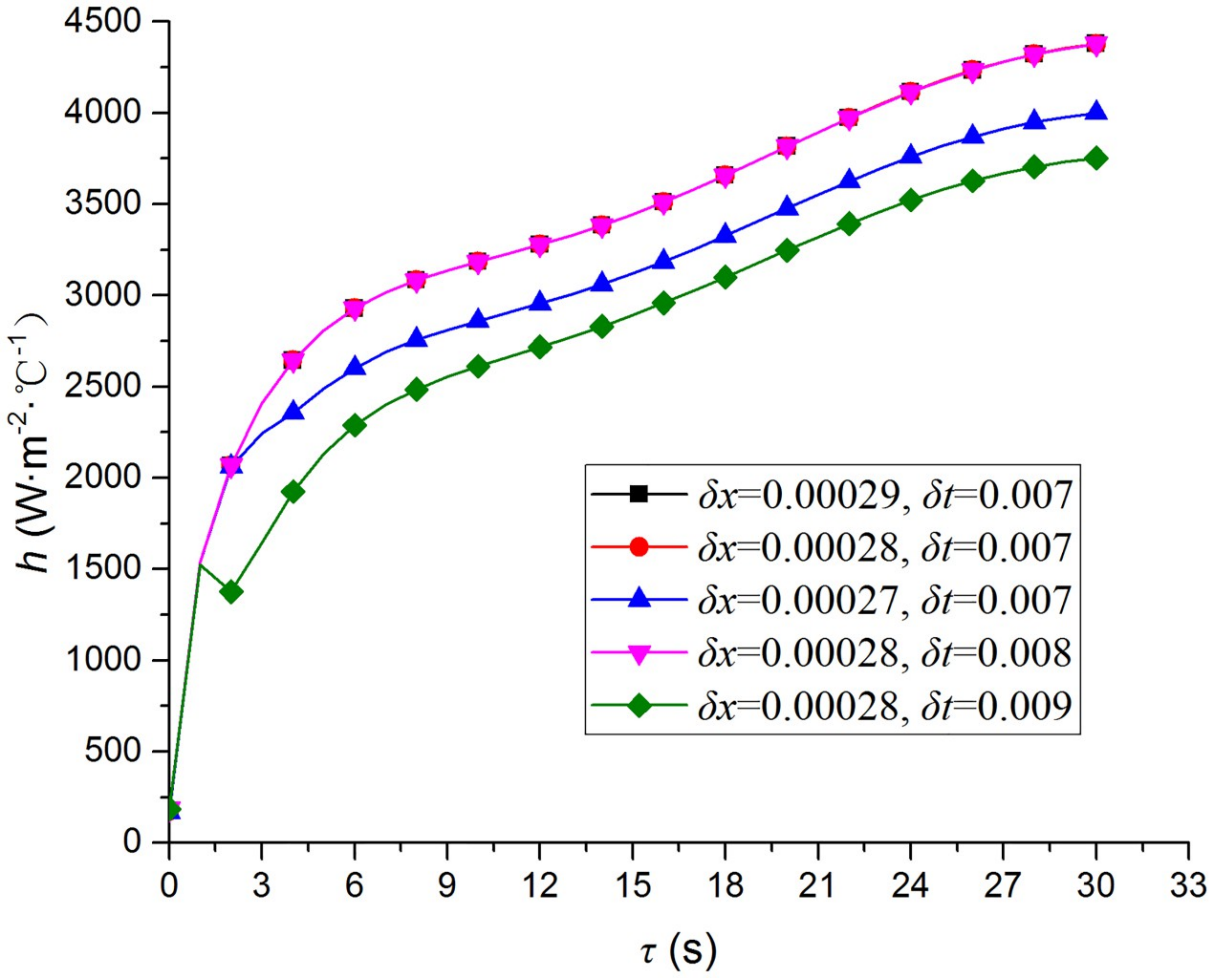
Time step and space step independence tests.

The convective heat transfer coefficients of TP1 at *H*/*D* = 4 are used to verify the independence of the time step and space step. Fig 8 shows that the grid system with *δx* = 0.028 and *δt* = 0.008 is accurate enough to guarantee the reliability of the numerical calculations and, therefore, it is adopted in the present study.

### Uncertainty analysis

In this study, the uncertainty of the measured impingement surface temperature *t*_*s*_ is estimated to be ±5%, including the uncertainty caused by the accuracy of the infrared camera (±3%), the uncertainty caused by the material emissivity test (±1.5%) [33], and other uncertainties (±0.5%). The uncertainty of the air jet temperature *t*_∞_ is about ±3%. The density, specific heat, and thermal conductivity have uncertainties of ±2%, ±2%, and 3%, respectively [28].

A statistical approach is used to estimate the propagation of the experimental uncertainties using a mathematical solution [34]. According to Eqs (3), (4) and (11), the convective heat transfer coefficient at a specific time can be expressed as a function of all the model parameters:

$$h=F(p_{1},p_{2},\ldots p_{l},\ldots p_{m})=F(\rho ,c_{p},\lambda ,t_{s},t_{\infty })$$ (17)

where *p*_*l*_ is a representative parameter, and *m* is the total number of relevant model parameters. Assuming that the value of each parameter is independently associated with a certain uncertainty interval *σ*, Eq (17) can be written as:

$$\begin{array}{ll} h\pm \sigma h & =F(p_{1}\pm \sigma p_{1},p_{2}\pm \sigma p_{2},\ldots p_{l}\pm \sigma p_{l},\ldots p_{m}\pm \sigma p_{m})\\ & =F(\rho \pm \sigma \rho ,c_{p}\pm \sigma c_{p},\lambda \pm \sigma \lambda ,t_{s}\pm \sigma t_{s},t_{\infty }\pm \sigma t_{\infty }) \end{array}$$ (18)

where *σh* denotes a probability distribution similar to the uncertainty probability distribution of all the primitive parameters and can be calculated as follows [34]:

$$\sigma h=\sqrt{\sum_{l=1}^{m}{\left(\frac{\partial F}{\partial pl}\sigma pl\right)}^{2}}$$ (19)

Let another solution of *h*_*l*_ be obtained at the same time. Using the actual values of all parameters with only one exception (the value of parameter *l* is as assumed to be *p*_*l*_ + *σp_l_*), the second solution is defined as:

$$h_{l}^{}=F(p_{1},p_{2},\ldots p_{l}+\sigma p_{l},\ldots p_{m})$$ (20)

The partial derivative in Eq (19) becomes:

$$\frac{\partial F}{\partial p_{l}}\sigma p_{l}≅\frac{h_{l}-h}{\Delta p_{l}}\sigma p_{l}$$ (21)

By selecting Δ*p*_*l*_ to be equal to *σp*_*l*_, Eq (21) can be written as:

$$\frac{\partial F}{\partial p_{l}}\sigma p_{l}≅h_{l}-h$$ (22)

In order to evaluate the influence of the uncertainty of each experimental parameter, the relative uncertainty (*RU*) can be defined as:

$$RU=\pm \left|\frac{h_{{}_{dev,*}}^{}-h}{h}\right|\times 100\%$$ (23)

where *h*_*dev*,*_, which may be *h*_*dev*,*ρ*_, *h*_*dev*,*λ*_, $h_{dev,c_{p}}$, $h_{dev,t_{\infty }}$, or $h_{dev,t_{s}}$, represents the convective heat transfer coefficient when one of the experimental parameters is consider to possess an artificial deviation according to its range of uncertainty. For example, *h*_*dev*,*ρ*_ represents the convective heat transfer coefficient when the density was added artificial deviation.

The total relative uncertainty (*RU*_*total*_) of the heat transfer coefficients is defined as:

$$RU_{total}=\pm \frac{\sigma h}{h}\times 100\%$$ (24)

where *RU*_*total*_ denotes the probability of the relative uncertainty of the heat transfer coefficients for the uncertainty probability distribution of all the primitive parameters.

**Fig 9:**
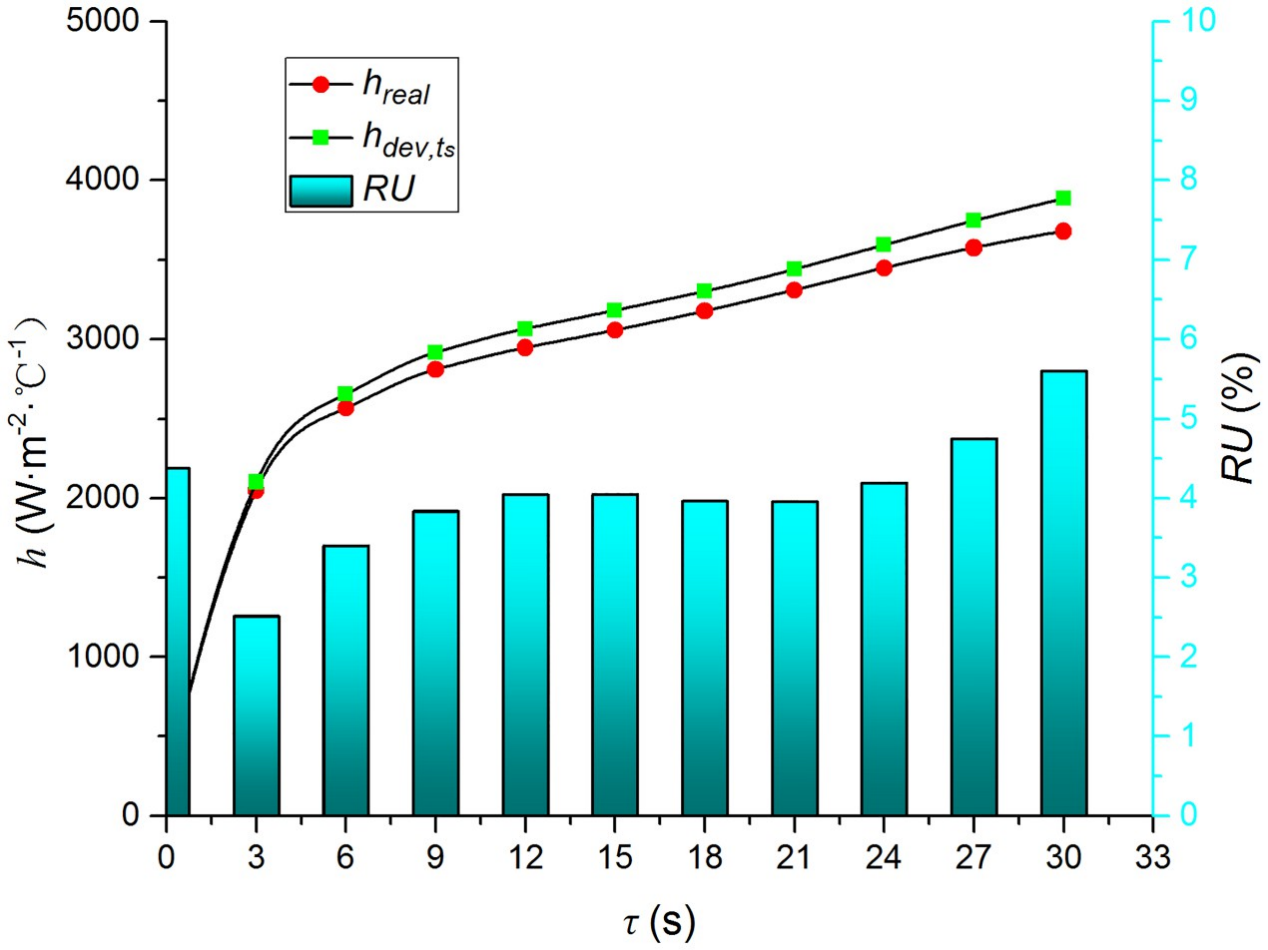
Comparison of $h_{dev,t_{s}}$ and *h*.

**Fig 10:**
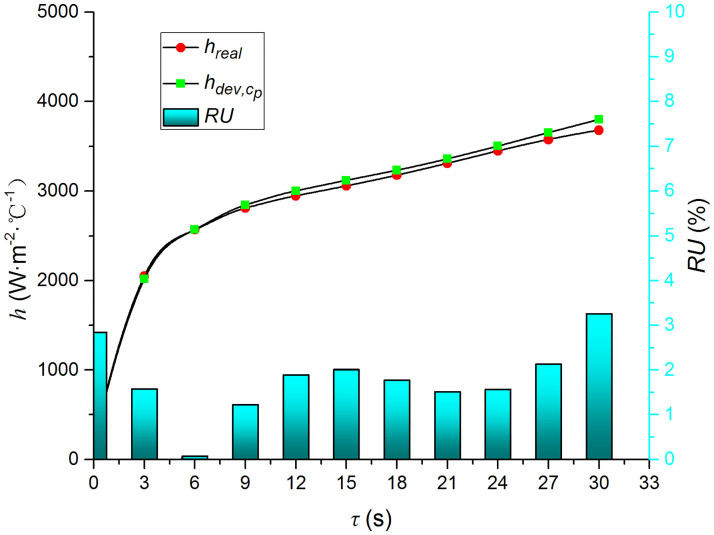
Comparison of $h_{dev,c_{p}}$ and *h*.

**Fig 11:**
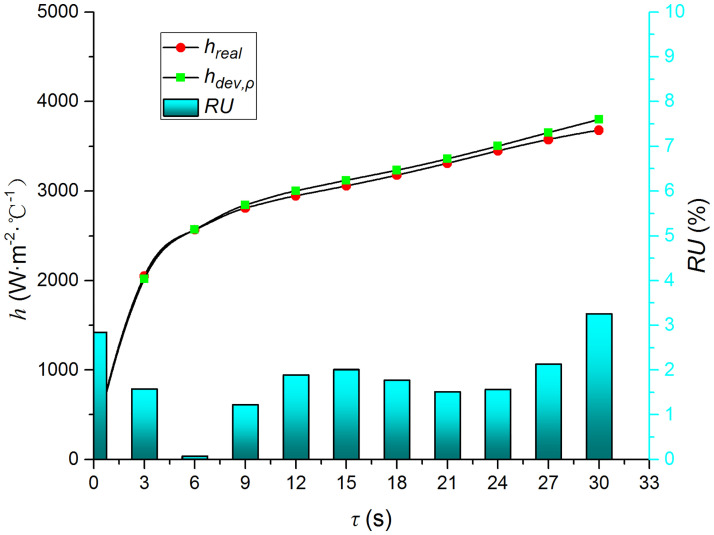
Comparison of *h*_*dev*,*ρ*_ and *h*.

**Fig 12:**
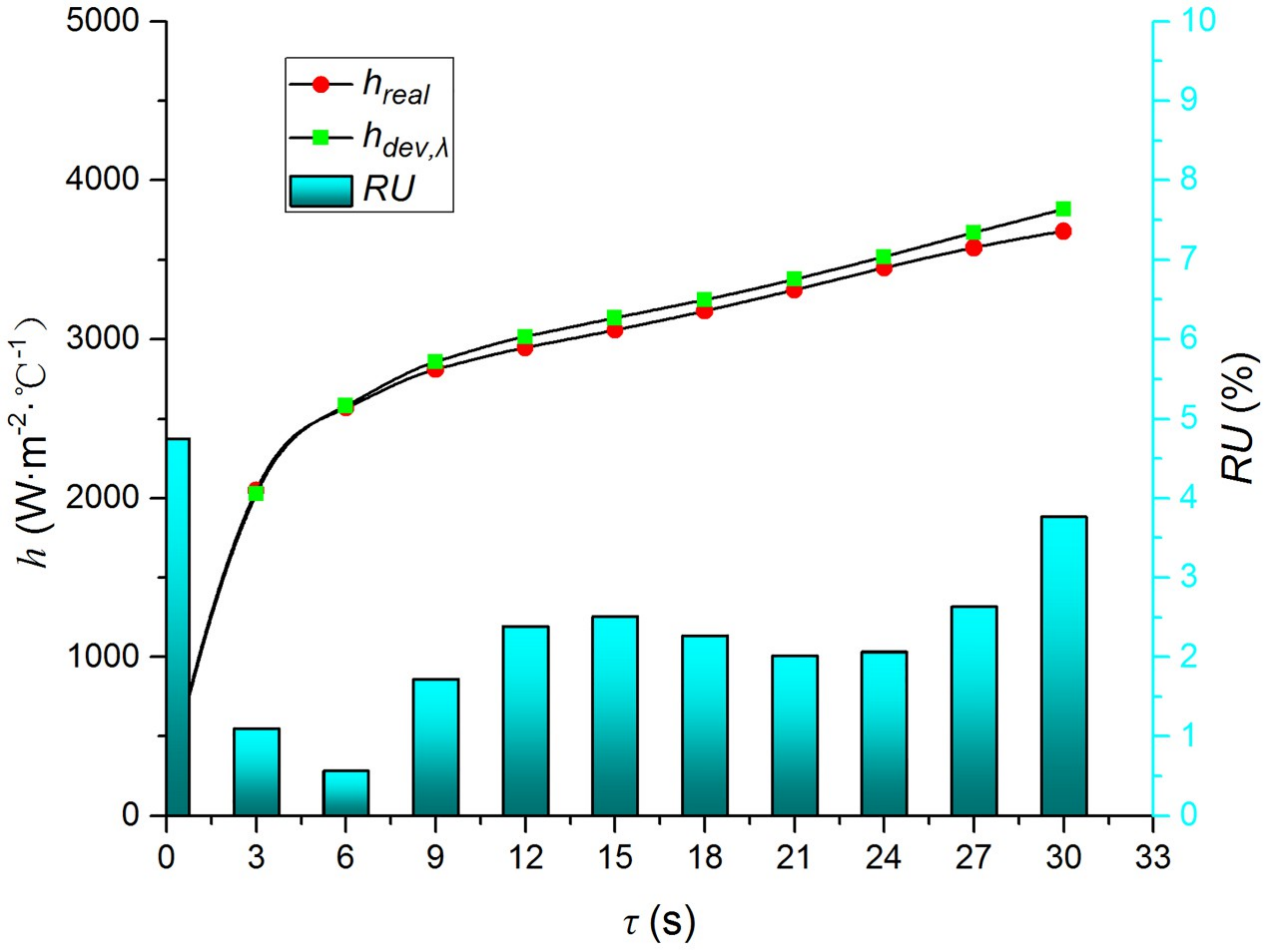
Comparison of *h*_*dev*,*λ*_ and *h*.

**Fig 13:**
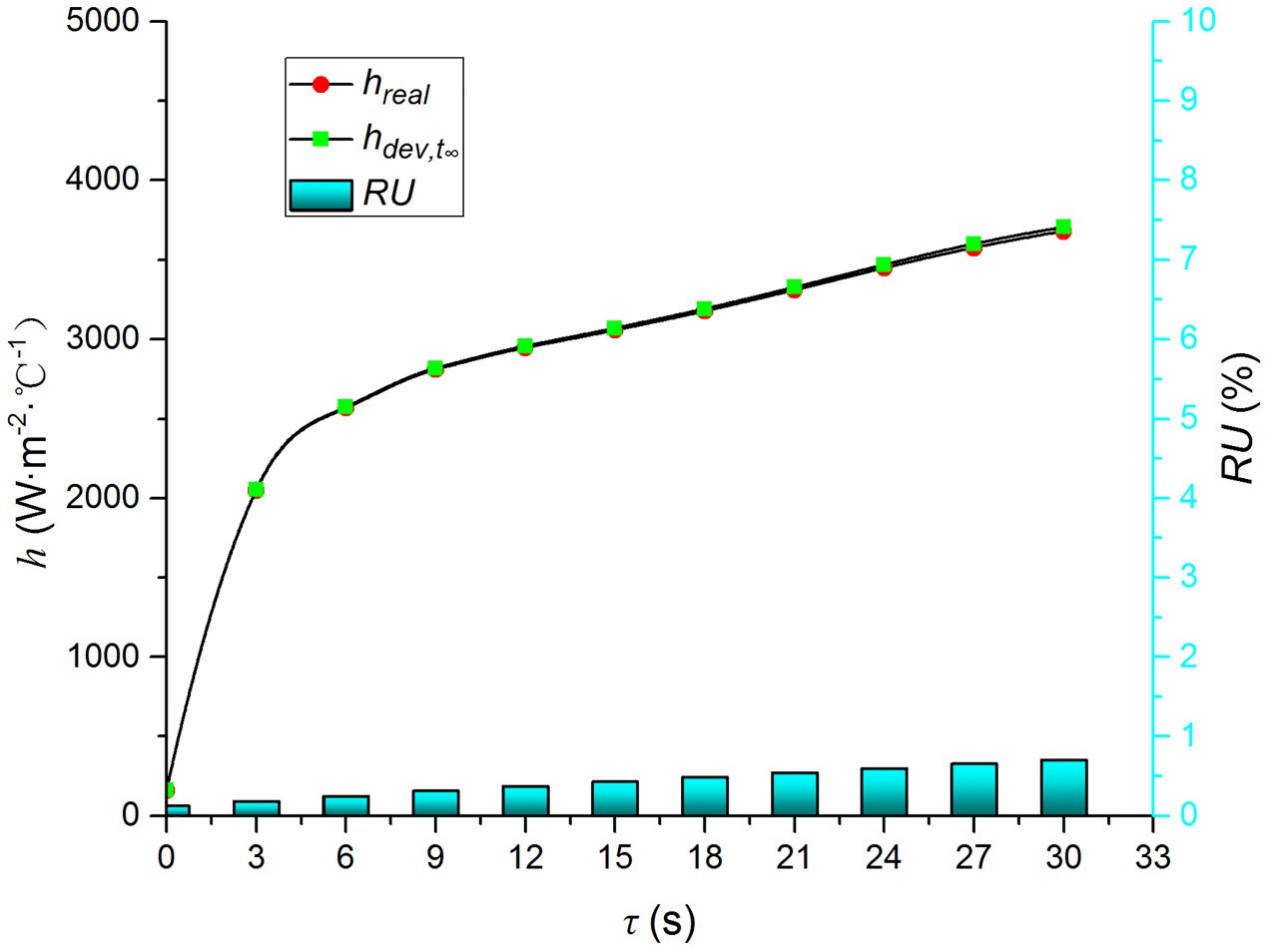
Comparison of $h_{dev,t_{\infty }}$ and *h*.

**Fig 14:**
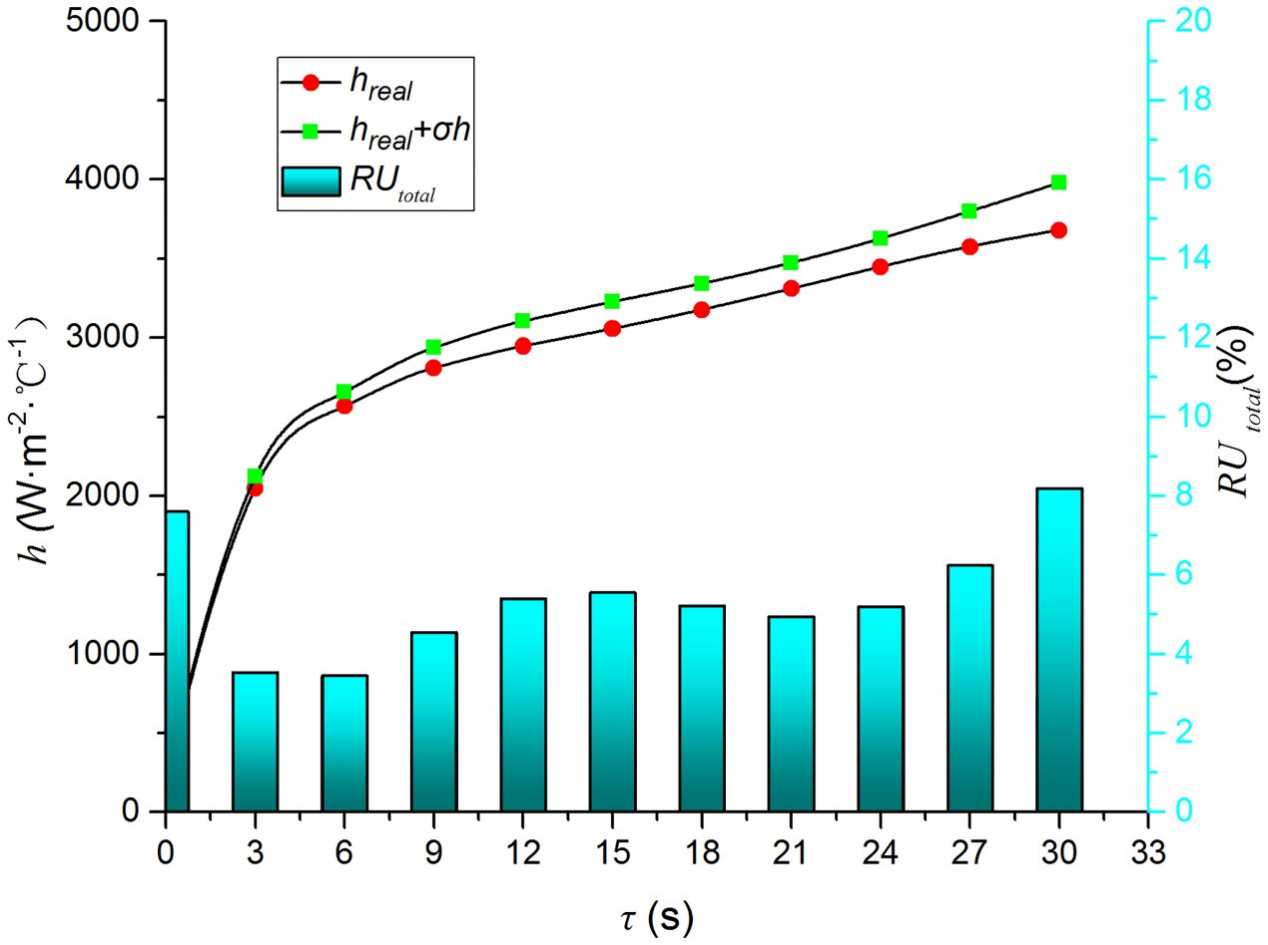
Comparison of *σh* and *h*.

Figs 9–14 compare the heat transfer coefficient and *RU* of MP1 at *H/D* = 3. The maximum relative uncertainties of the convective heat transfer coefficient caused by the measured temperature uncertainty (±5%), density uncertainty (±2%), specific heat capacity uncertainty (±2%), and thermal conductivity uncertainty (±3%) are ±5.59%, ±3.25%, ±3.25%, and ±4.74%, respectively. The influence of the air jet temperature cannot be ignored; it affects also the numerical results. The lower the impingement surface temperature, the more pronounced the influence of the ambient temperature. The total relative uncertainty of the heat transfer coefficients is less than ±8.1%.

## Results and discussion

### Effect of the thermophysical parameters

As shown in the heat transfer network of Fig 1, the jet impingement heat transfer is a conjugate process, which includes the internal heat conduction of solid domains. In addition, the thermophysical parameters, such as the density, specific heat capacity, and thermal conductivity, of the solid domain are significantly influenced by the temperature. In order to quantitatively analyze the effect of the aforementioned three parameters on the heat transfer characteristics, the relative error (*RE*) of the convective heat transfer coefficients with different thermophysical parameters is analyzed; it can be defined as:

$$RE=\left|\frac{h_{con,*}-h_{\text{var}\;y}}{h_{\text{var}\;y}}\right|\times 100\%$$ (25)

where *h*_*con*,*_, which may be *h*_*con*,*ρ*_, *h*_*con*,*λ*_, $h_{con,c_{p}}$, or *h*_*con*,*all*_, represents the convective heat transfer coefficient when one or all of the three parameters are constant. For example, *h*_*con*,*ρ*_ represents the convective heat transfer coefficient when the density is constant, and *h*_*con*,*all*_ represents the convective heat transfer coefficient when all parameters are constant. Furthermore, *h*_var *y*_ represents the convective heat transfer coefficient when all three parameters are vary with temperature.

**Fig 15:**
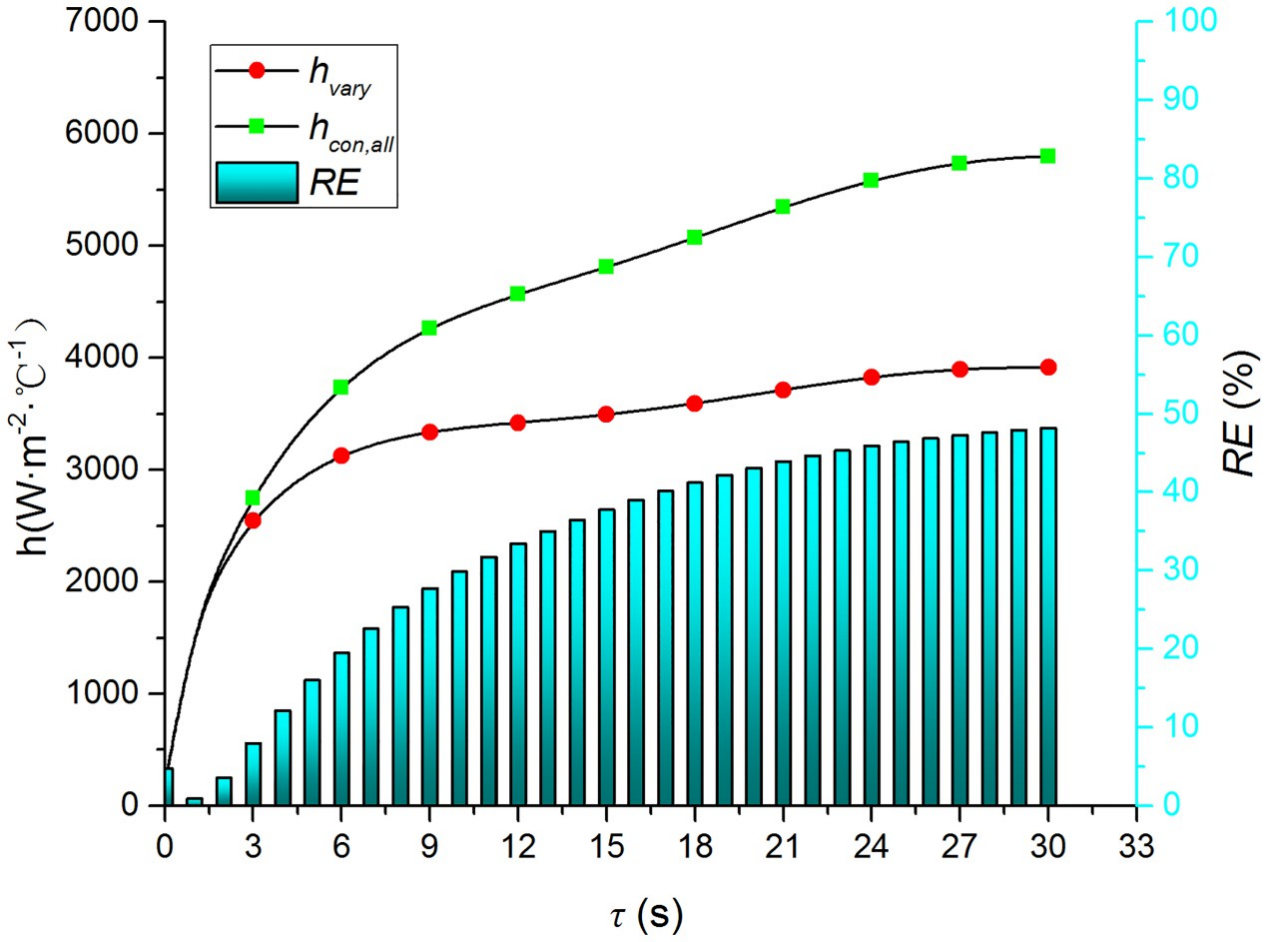
Comparison of *h*_*con*,*all*_ and *h*_var *y*_.

**Fig 16:**
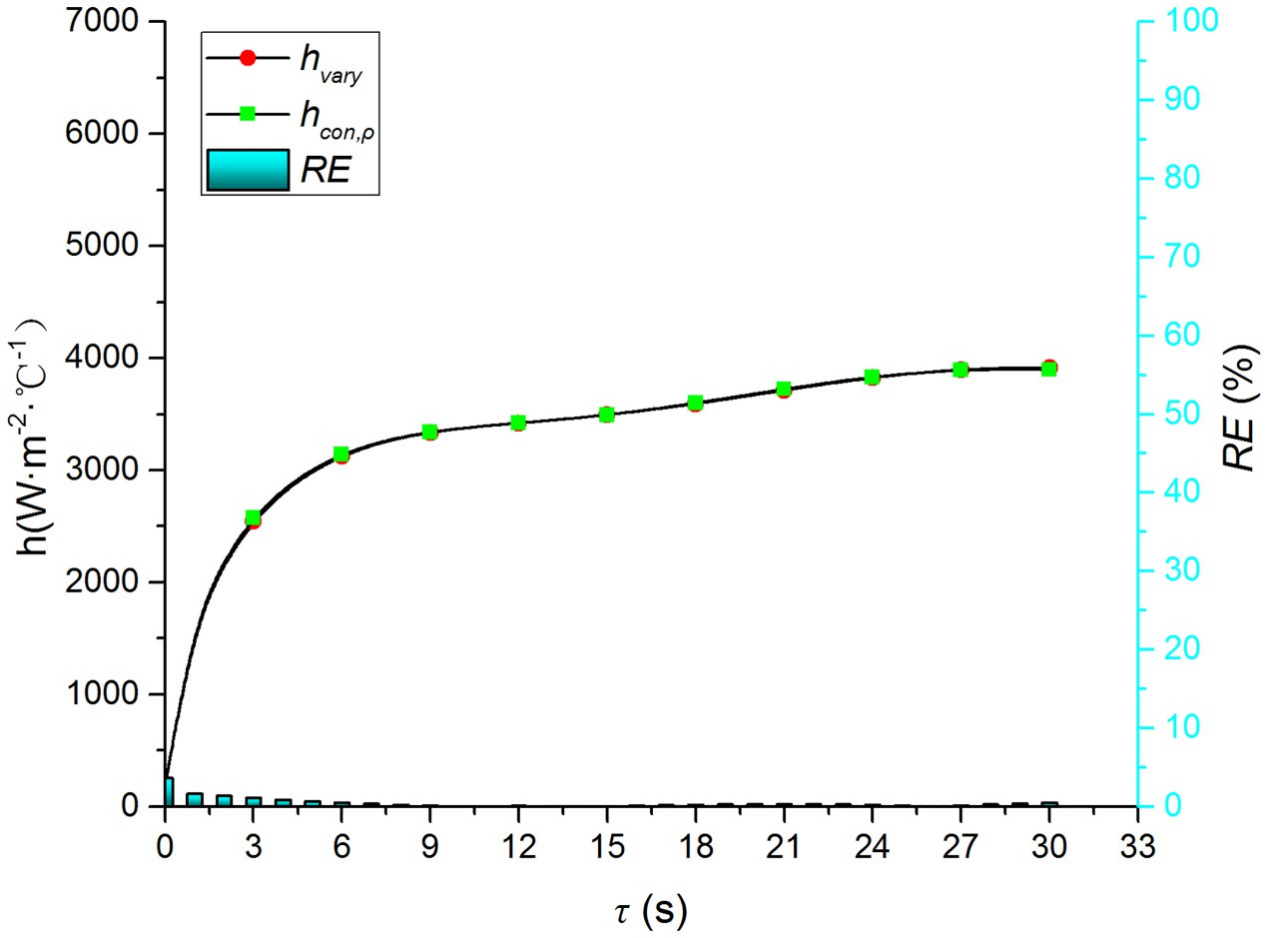
Comparison of *h*_*con*,*ρ*_ and *h*_var *y*_.

**Fig 17:**
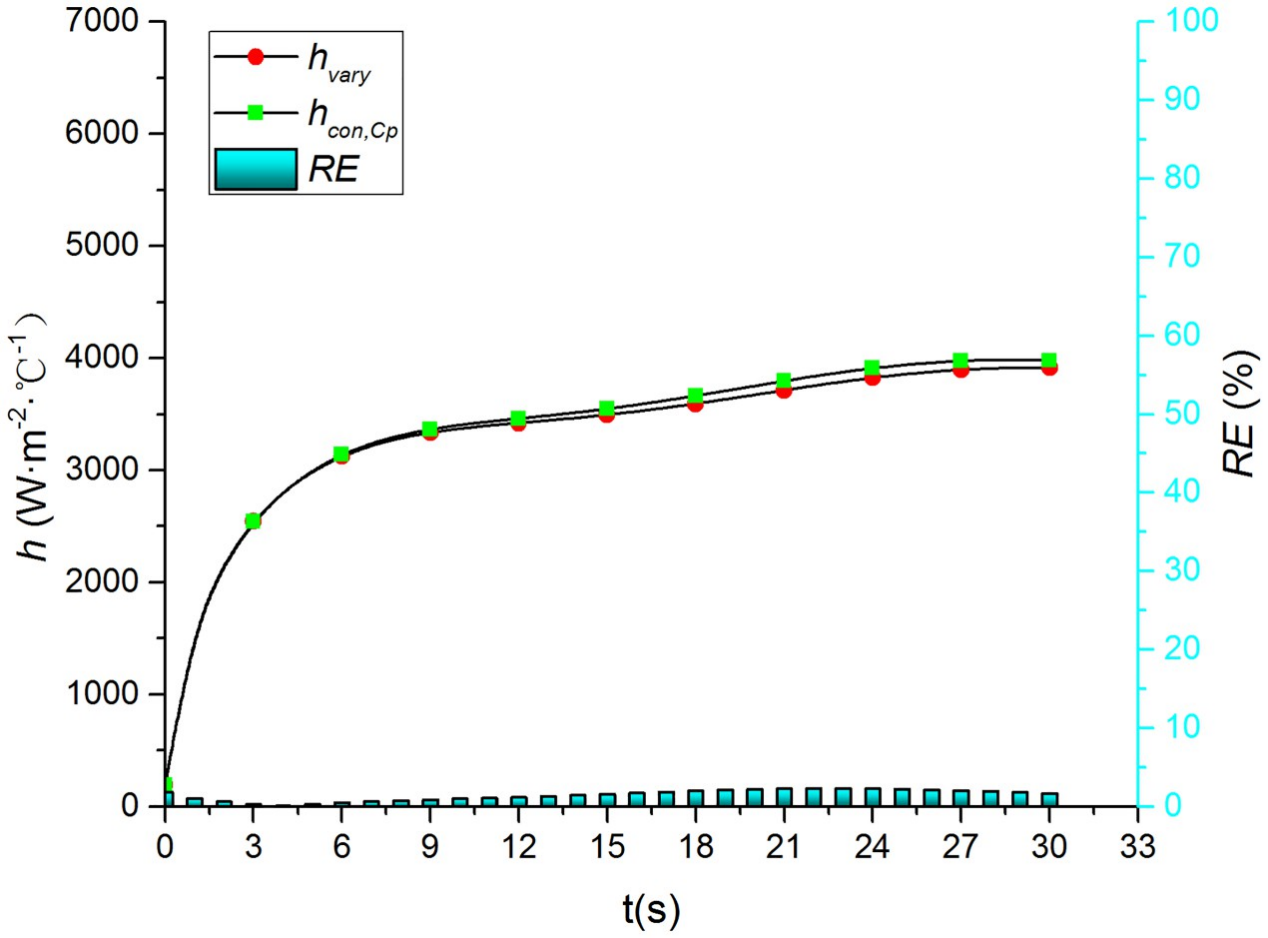
Comparison of $h_{con,c_{p}}$ and *h*_var *y*_.

**Fig 18:**
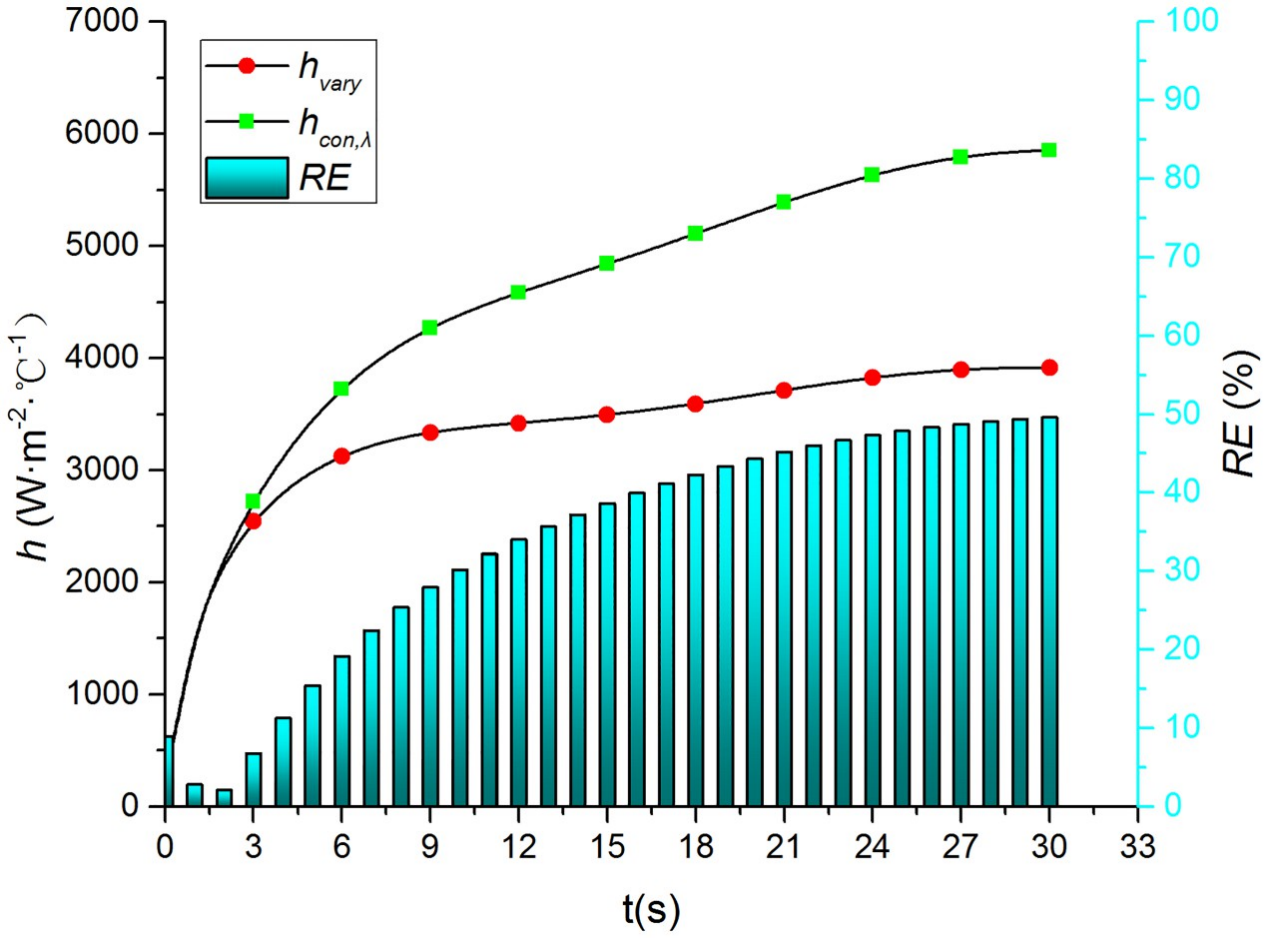
Comparison of *h*_*con*,*λ*_ and *h*_var *y*_.

Figs 15–18 compare the convective heat transfer coefficient and *RE* of MP1 at *H/D* = 5. It can be seen that the variation in the thermophysical parameters with temperature will significantly affect the predicted heat transfer coefficient. The *RE* between *h*_*con*,*all*_ and *h*_var *y*_ increases gradually and reaches the maximum value of 49.6 at the end of the jet impingement. The main source of error is the thermal conductivity, and the maximum *RE* between *h*_*con*,*λ*_ and *h*_var *y*_ is found to be 48.18. The density and specific heat capacity have a limited impact on the results, and the maximum *RE* values are found to be 3.62 and 2.28 when either the density or the specific heat capacity are kept constant, respectively. Therefore, it is necessary to consider the variation in the thermophysical parameters with temperature when studying the transient jet impingement heat transfer.

### Heat transfer of the supersonic air jet impingement on a high-temperature target

**Fig 19:**
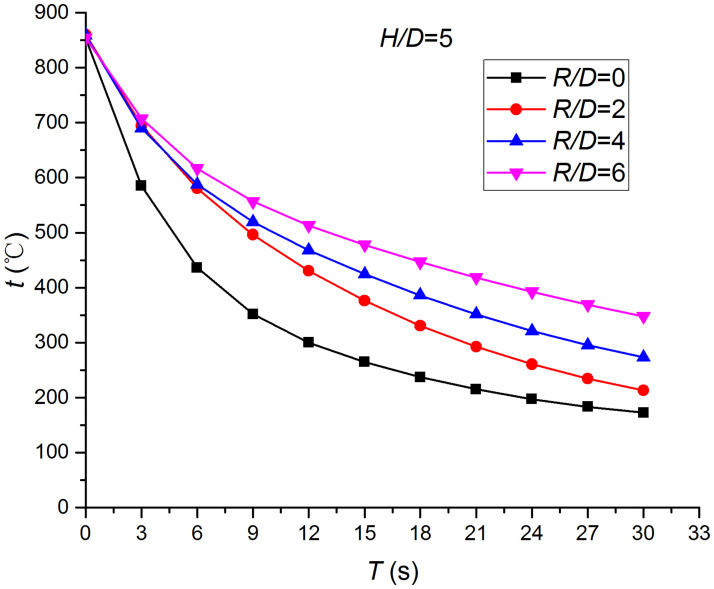
Distribution and variation of the surface temperature.

**Fig 20:**
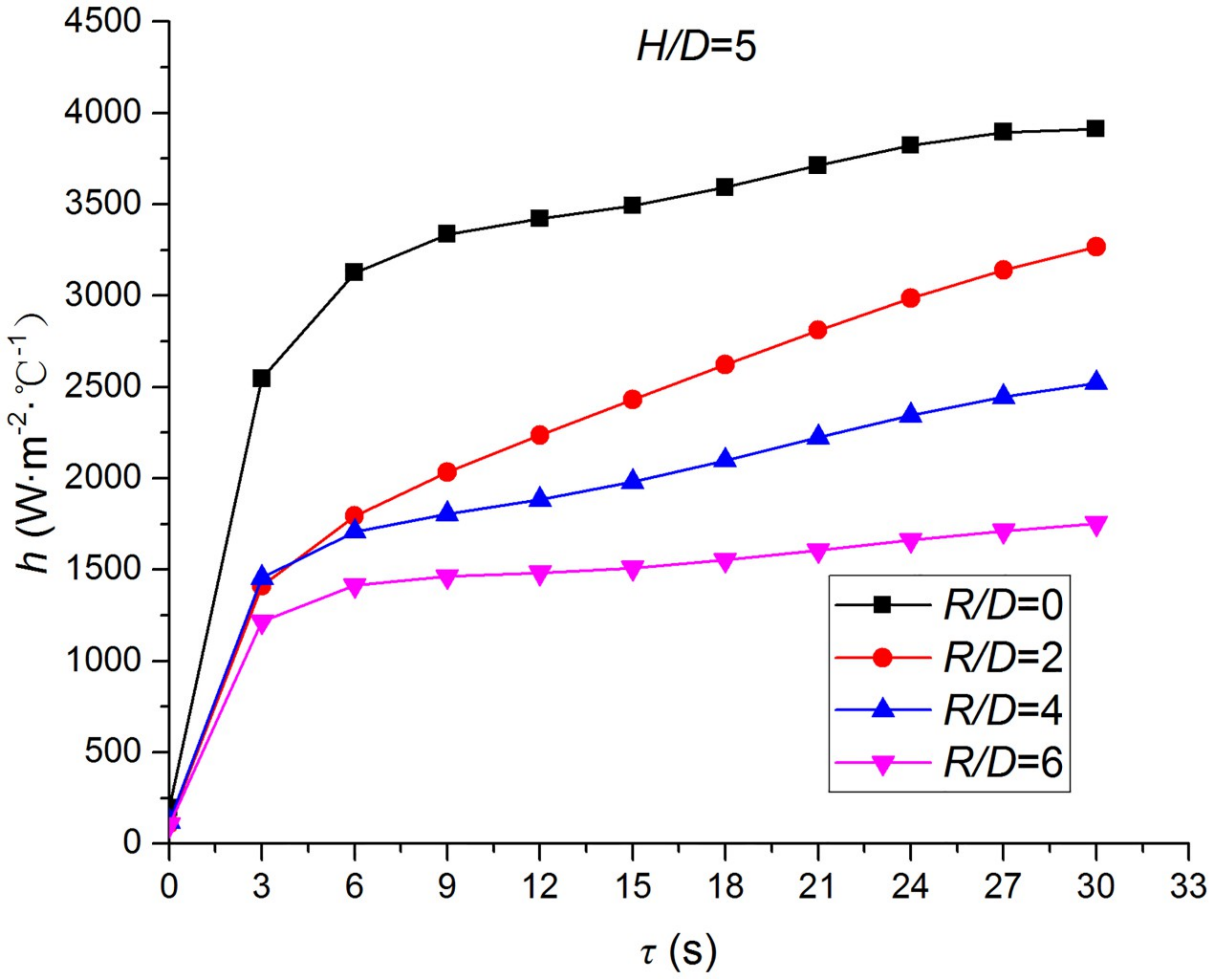
Distribution and variation of the convective heat transfer coefficient.

**Fig 21:**
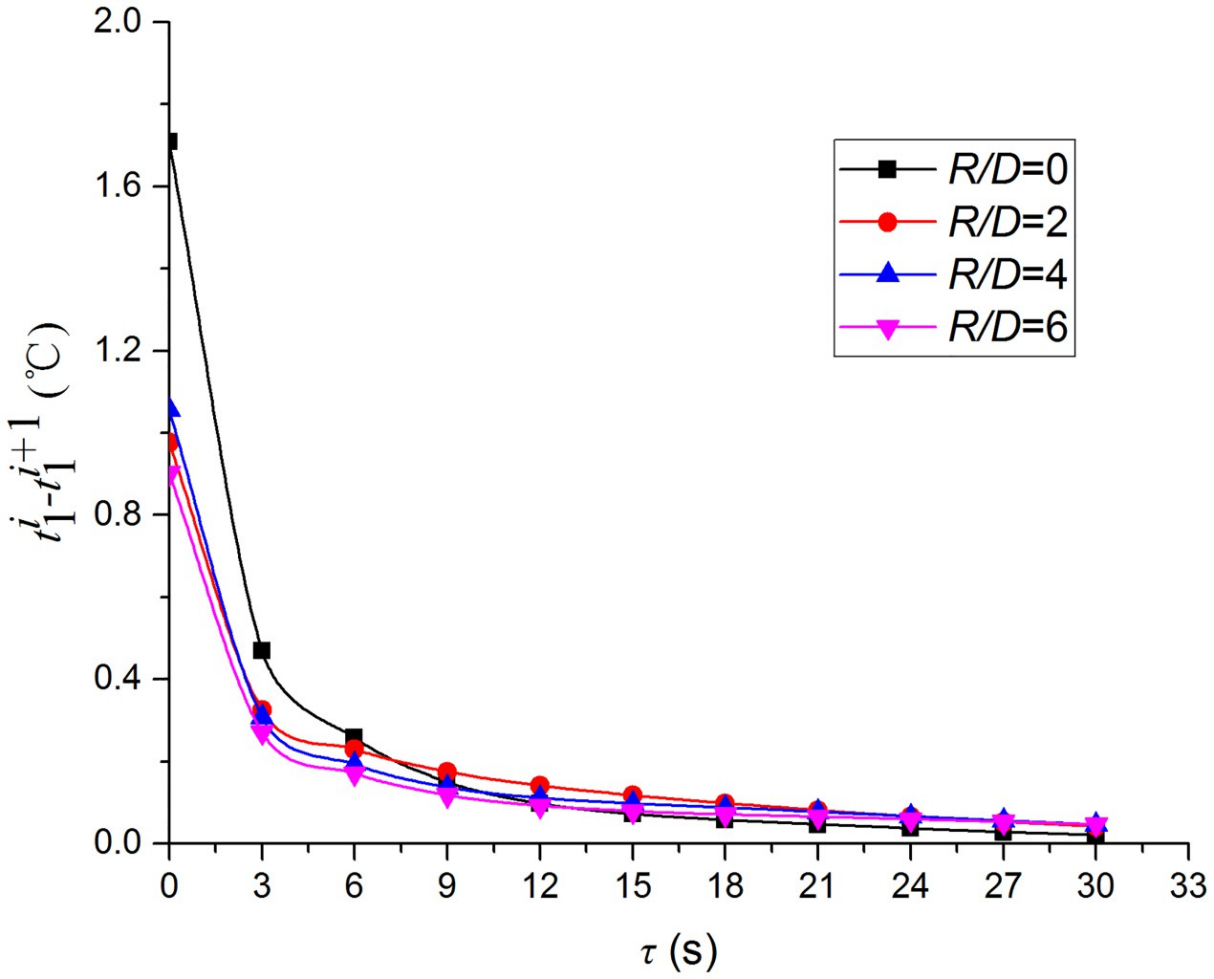
Variations of $\left(t_{1}^{i}-t_{1}^{i+1}\right)$.

**Fig 22:**
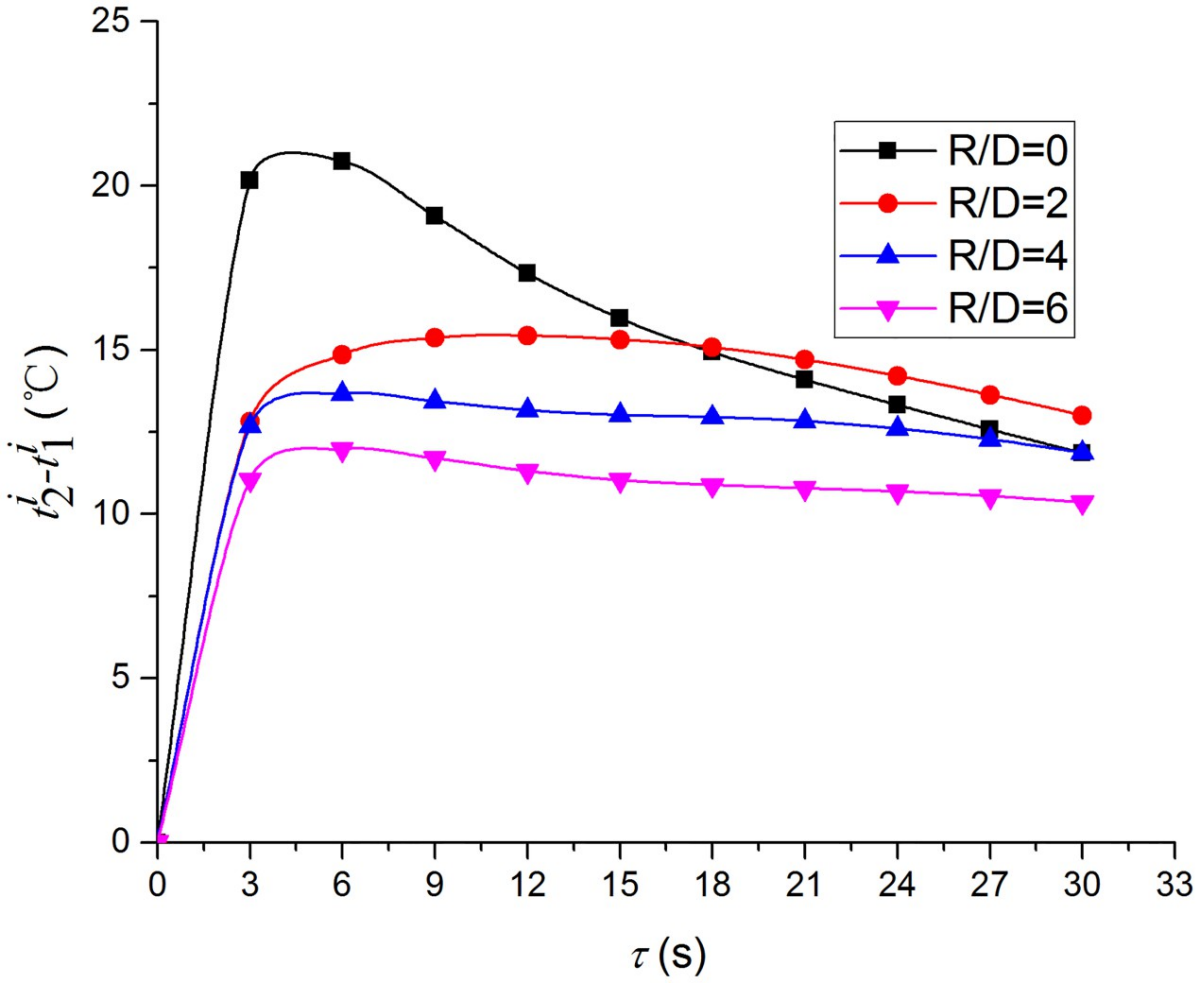
Variations of $\left(t_{2}^{i}-t_{1}^{i}\right)$.

Figs 19 and 20 depict the distribution and variation of the surface temperature and convective heat transfer coefficient at *H/D* = 5, respectively. Where *R/D* and *H/D* represent the nondimensional distance of the test position and the nondimensional nozzle-to-target distance ratio, respectively. It can be observed that the surface temperature decreases rapidly at the beginning of the impingement, and this trend slows down after 3 s. Additionally, the convective heat transfer coefficient increases rapidly at the beginning of the impingement, and this trend also slows down after 3 s. In order to analyze the conjugate heat transfer characteristics of the supersonic air jet impingement in detail, Figs 21 and 22 present the variation in the temperature difference of the heat transfer surface in a time step $\left(t_{1}^{i}-t_{1}^{i+1}\right)$ and the temperature difference between the heat transfer surface and adjacent nodes $\left(t_{2}^{i}-t_{1}^{i}\right)$, respectively. It can be observed that the value of $\left(t_{1}^{i}-t_{1}^{i+1}\right)$ continues to decrease during the jet impingement.

The value of $\left(t_{2}^{i}-t_{1}^{i}\right)$ increases rapidly at the beginning of the jet impingement and becomes stable after 3 s. According to Eq (11), in addition to the thermophysical parameters, the convective heat transfer coefficient increases with the decrease in the temperature difference between the jet and the heat transfer surface $\left(t_{1}^{i}-t_{\infty }\right)$, decreases with the increase in $\left(t_{1}^{i}-t_{1}^{i+1}\right)$, and decreases with the increase in $\left(t_{2}^{i}-t_{1}^{i}\right)$. However, the variation in the convective heat transfer coefficient exhibits the opposite trend with $\left(t_{1}^{i}-t_{1}^{i+1}\right)$, which indicates that the value of $\left(t_{2}^{i}-t_{1}^{i}\right)$ greatly affects the heat transfer characteristics of the jet impingement. The value of $\left(t_{2}^{i}-t_{1}^{i}\right)$ is not only related to the heat transfer capacity of the jet but also to the size and thermophysical properties of the solid domain. Hence, the results are influenced by the type of material. Therefore, it is necessary to consider the influence of the solid domain when studying the heat transfer characteristics of the jet impingement.

**Fig 23:**
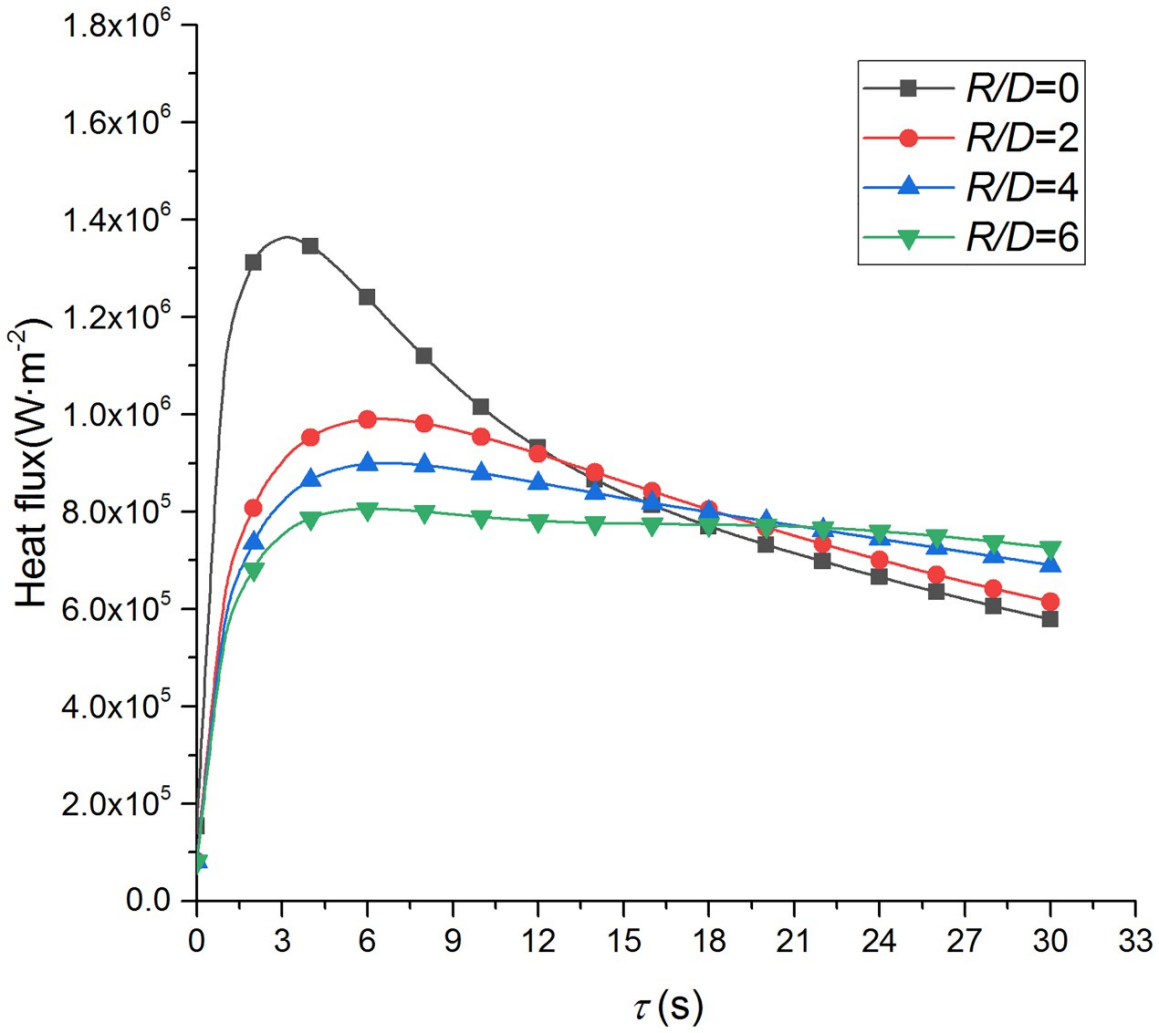
Heat flux of the supersonic air jet impingement.

**Fig 24:**
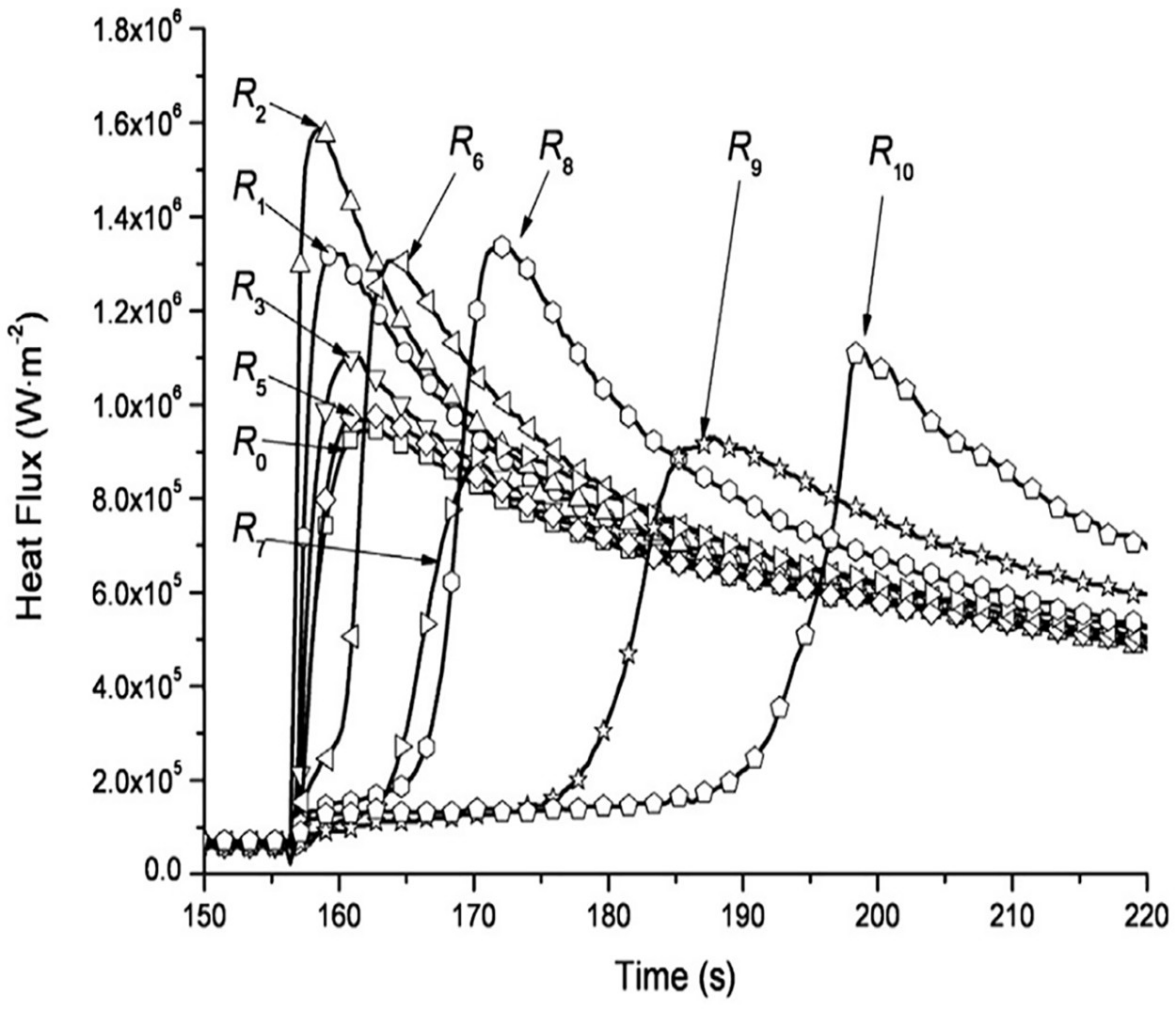
Heat flux of the water jet impingement.

In order to evaluate the heat transfer capacity of the supersonic air jet, Figs 23 and 24 compare the heat flux of the supersonic air jet and water jet. Fig 23 presents the heat flux of the supersonic air jet impingement of the present study, and the heat flux (*q*^*i*^) of the heat transfer surface at time *i* can be written as:

$$q^{i}=\rho c_{p}\frac{\delta x}{2}\frac{t_{1}^{i+1}-t_{1}^{i}}{\delta \tau }-\lambda \frac{t_{2}^{i}-t_{1}^{i}}{\delta x}\;i=1,\ldots ,\frac{T}{\delta \tau }$$ (26)

Fig 24 presents the heat flux of the water jet impingement on a high-temperature (around 950°C) stainless-steel plate [23]. The diameter of the nozzle is 7.5 mm, and the rate and pressure of the flow are 50 L/min and 2 MPa, respectively; *R*_0_ represents the center of the heat transfer interface, and *R*_1_ to *R*_10_ denote the *R/D* = 1, 2, 3, 4, 5, 6.5, 8, 9.5, 11, and 12.5 positions, respectively. It can be seen that the heat transfer capacity is greatly improved using a supersonic air jet and becomes even comparable to that of the water jet at some points.

### Effect of the nozzle-to-target distance

Figs 25–32 illustrate the distribution and variation of the surface temperature and convective heat transfer coefficient at *H/D* = 3, 4, and 5, respectively. In order to analyze the influence of the nozzle-to-target distance on the convective heat transfer coefficient, the average deviation (*AD*) can be written as:

$$AD=\frac{\sum_{i=0}^{T/\delta \tau -1}\left((h_{\text{max}}^{i}-h_{\text{min}}^{i})/h_{\text{max}}^{i}\right)}{T/\delta \tau }\times 100\%$$ (27)

where $h_{\text{max}}^{i}$ and $h_{\text{min}}^{i}$ represent the convective heat transfer coefficients at time *i* at the nozzle-to-target distance with the maximum and minimum average heat transfer coefficient, respectively. Table 3 illustrates the average convective heat transfer coefficient and *AD* at different heat transfer positions and nozzle-to-target distances. It can be seen that at the center of the heat transfer interface (*R/D* = 0), the temperature reduction rate and convective heat transfer coefficient are clearly affected by the nozzle-to-target distance. Hence, the convective heat transfer coefficient at *H*/*D* = 3 is significantly smaller than those at the other two distances, and *AD* = 13.77%. At the other locations (*R/D* = 2, 4, and 6), the convective heat transfer coefficient is less affected by the nozzle-to-target distance, and the *AD* values at *R/D* = 0, 2, 4, and 6 are found to be 0, 5.79%, 7.46%, and 6.29%, respectively.

**Fig 25:**
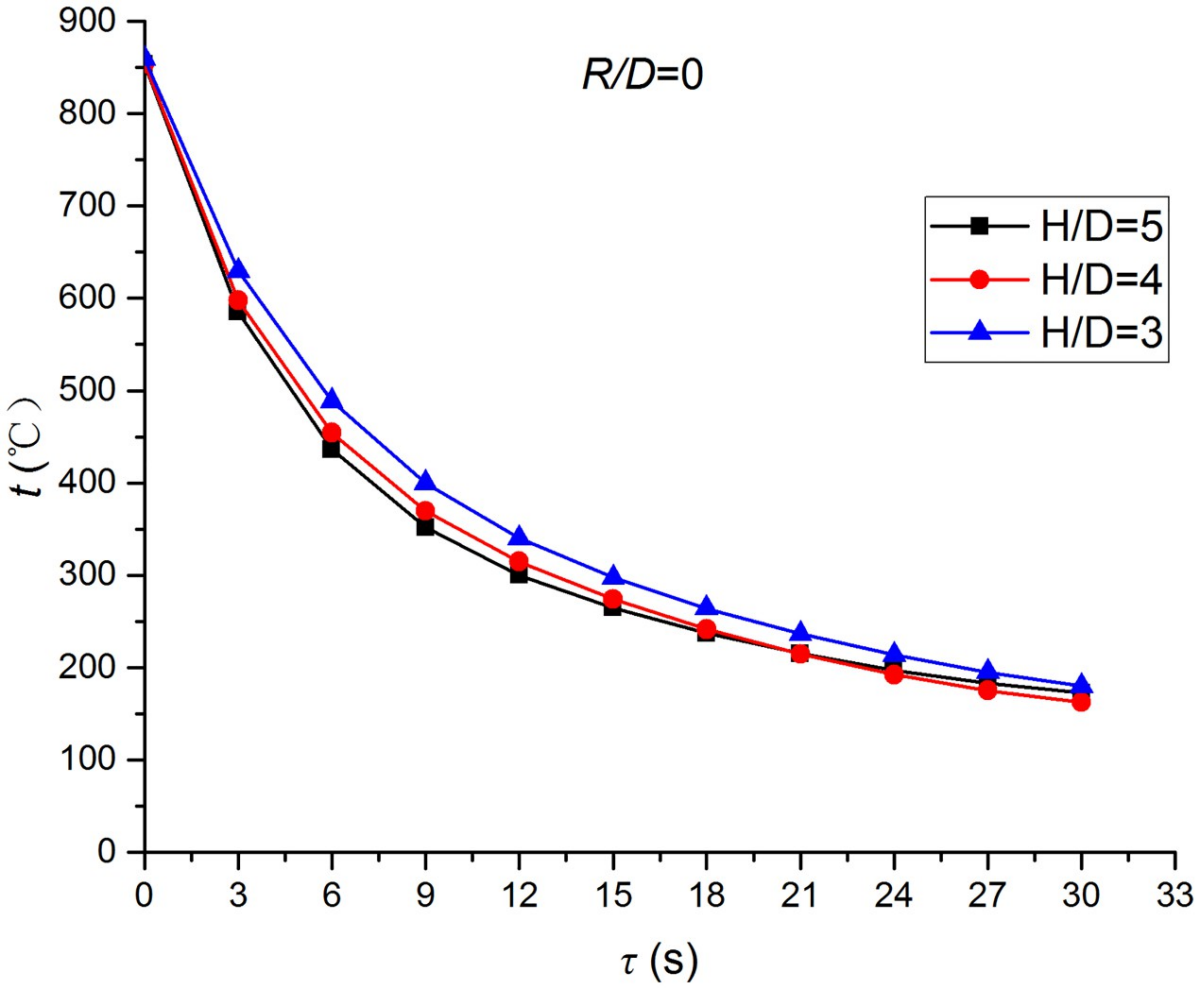
Surface temperature at *R/D* = 0.

**Fig 26:**
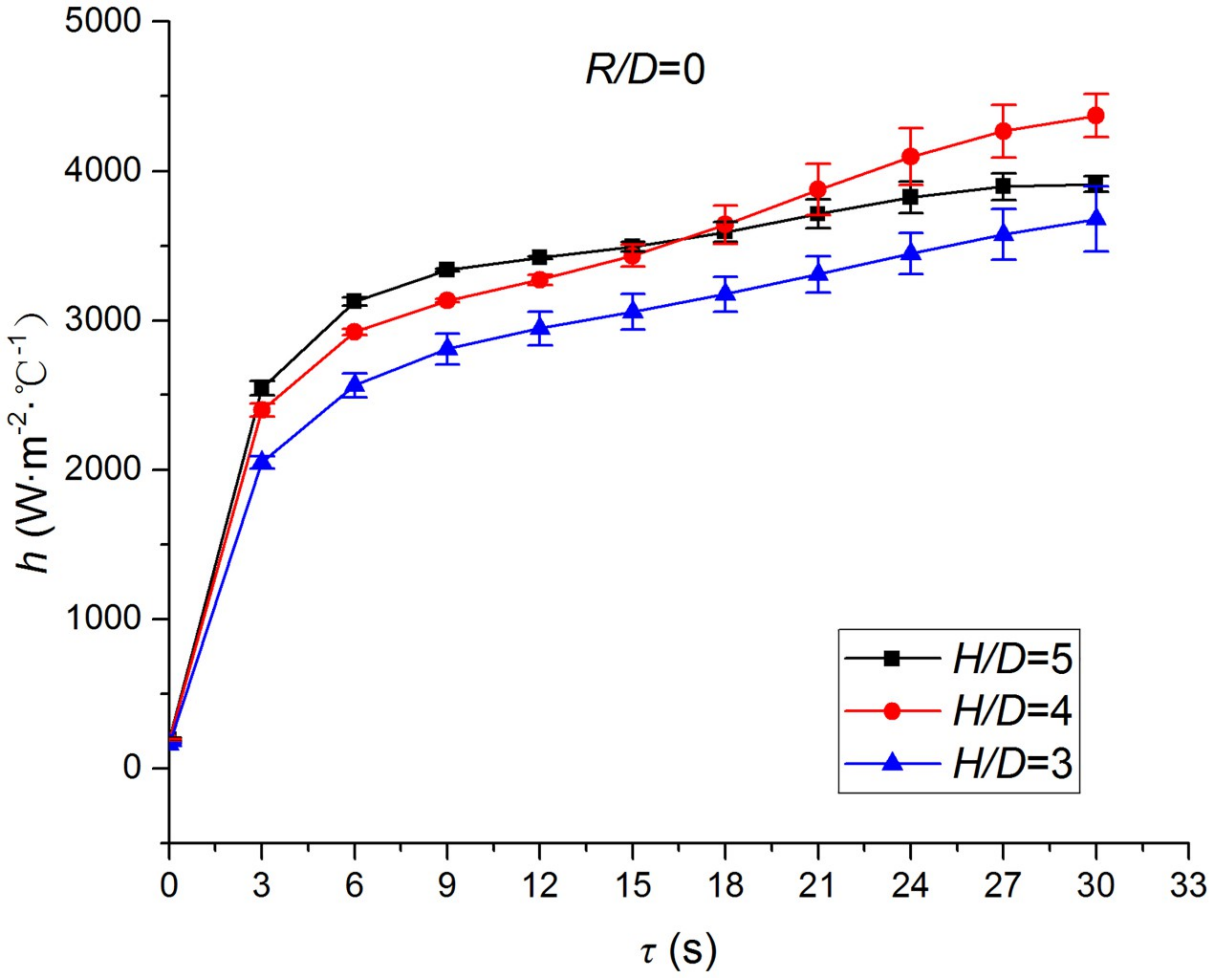
Convective heat transfer coefficient at *R/D* = 0.

**Fig 27:**
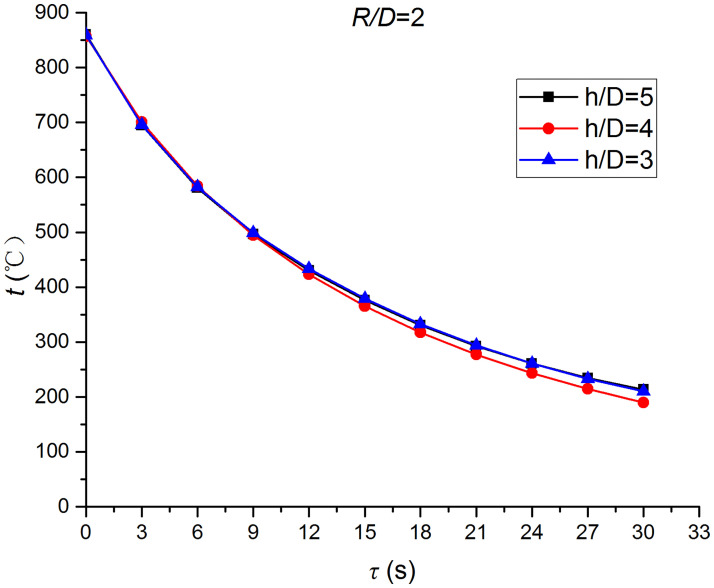
Surface temperature at *R/D* = 2.

**Fig 28:**
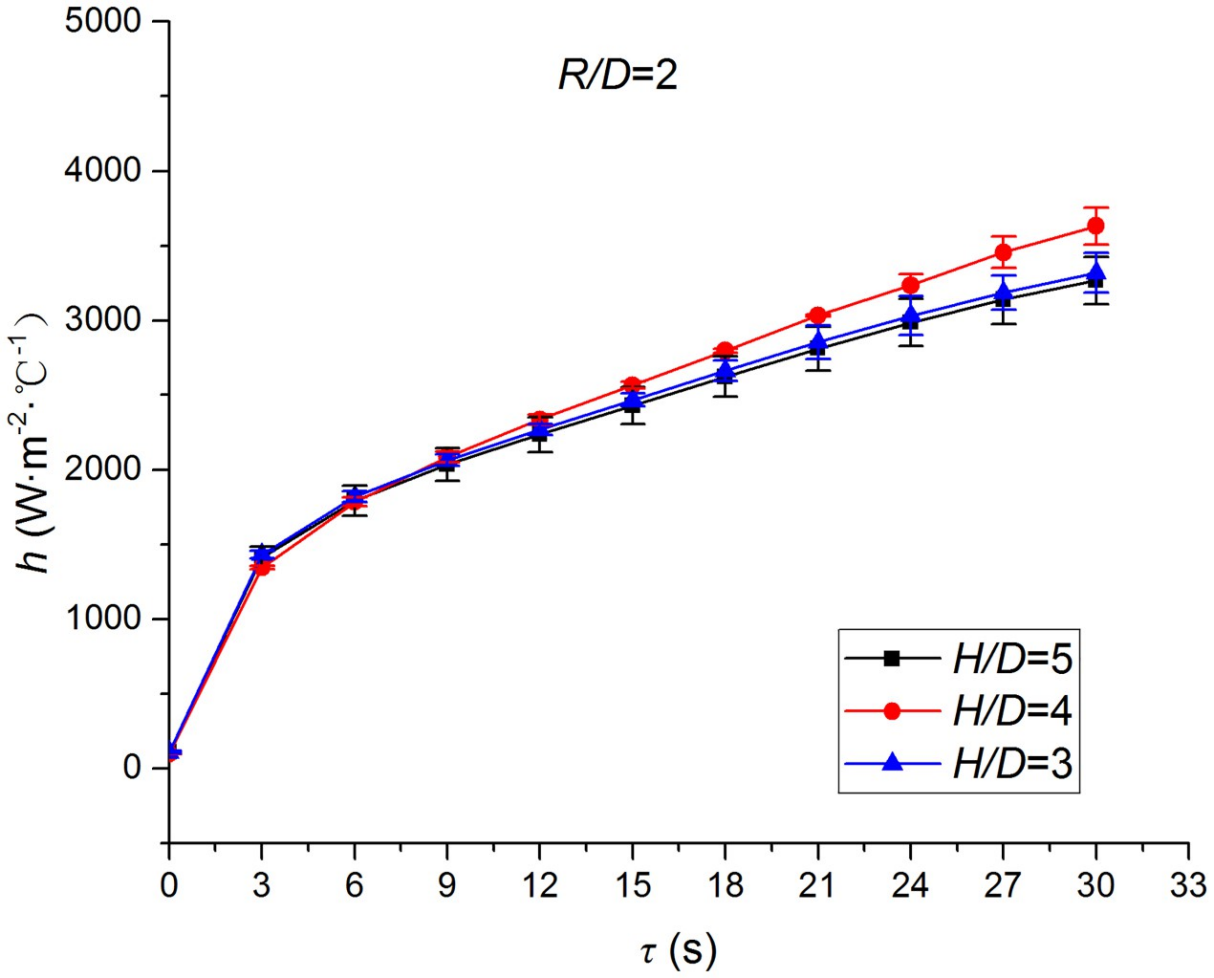
Convective heat transfer coefficient at *R/D* = 2.

**Fig 29:**
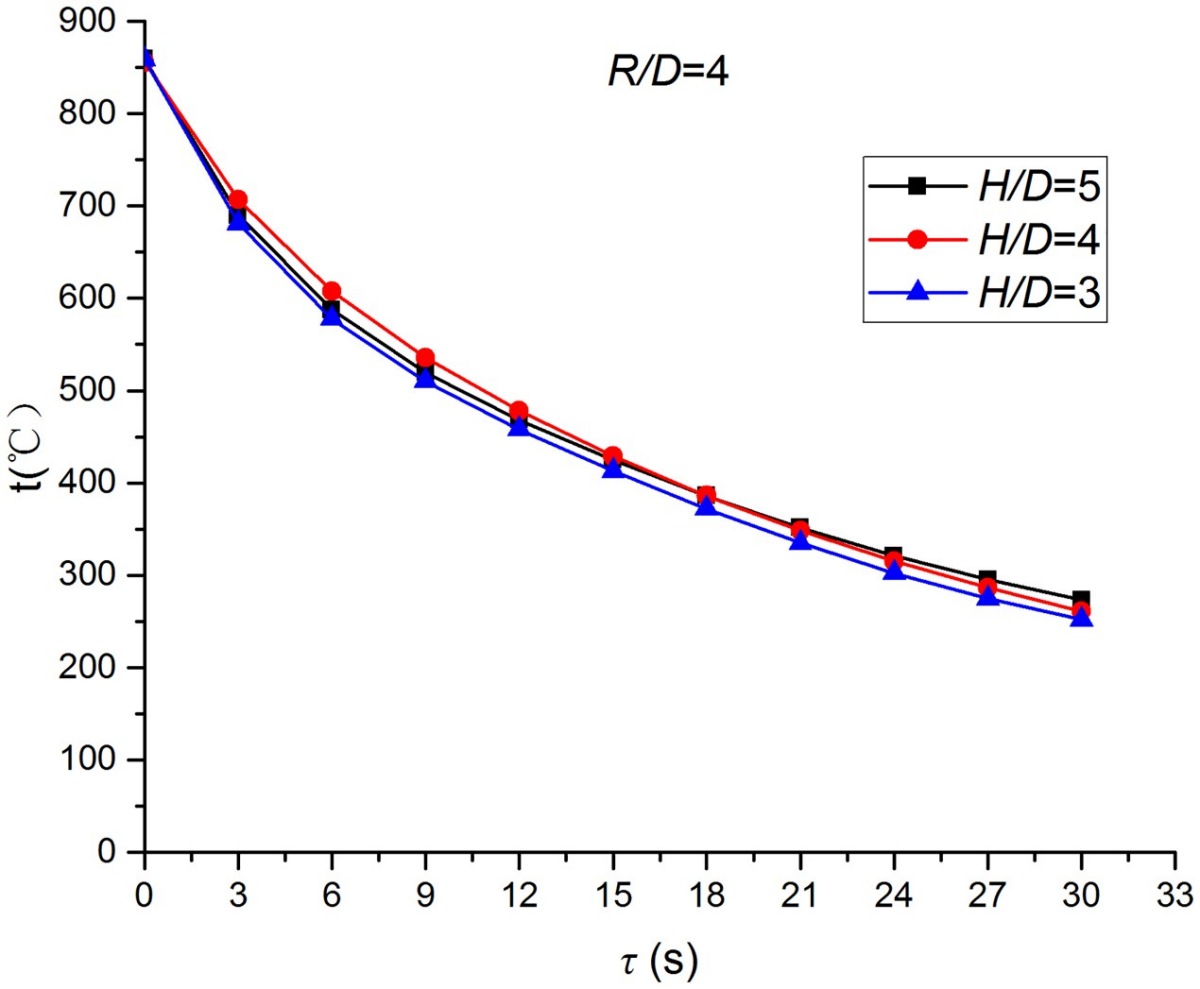
Surface temperature at *R/D* = 4.

**Fig 30:**
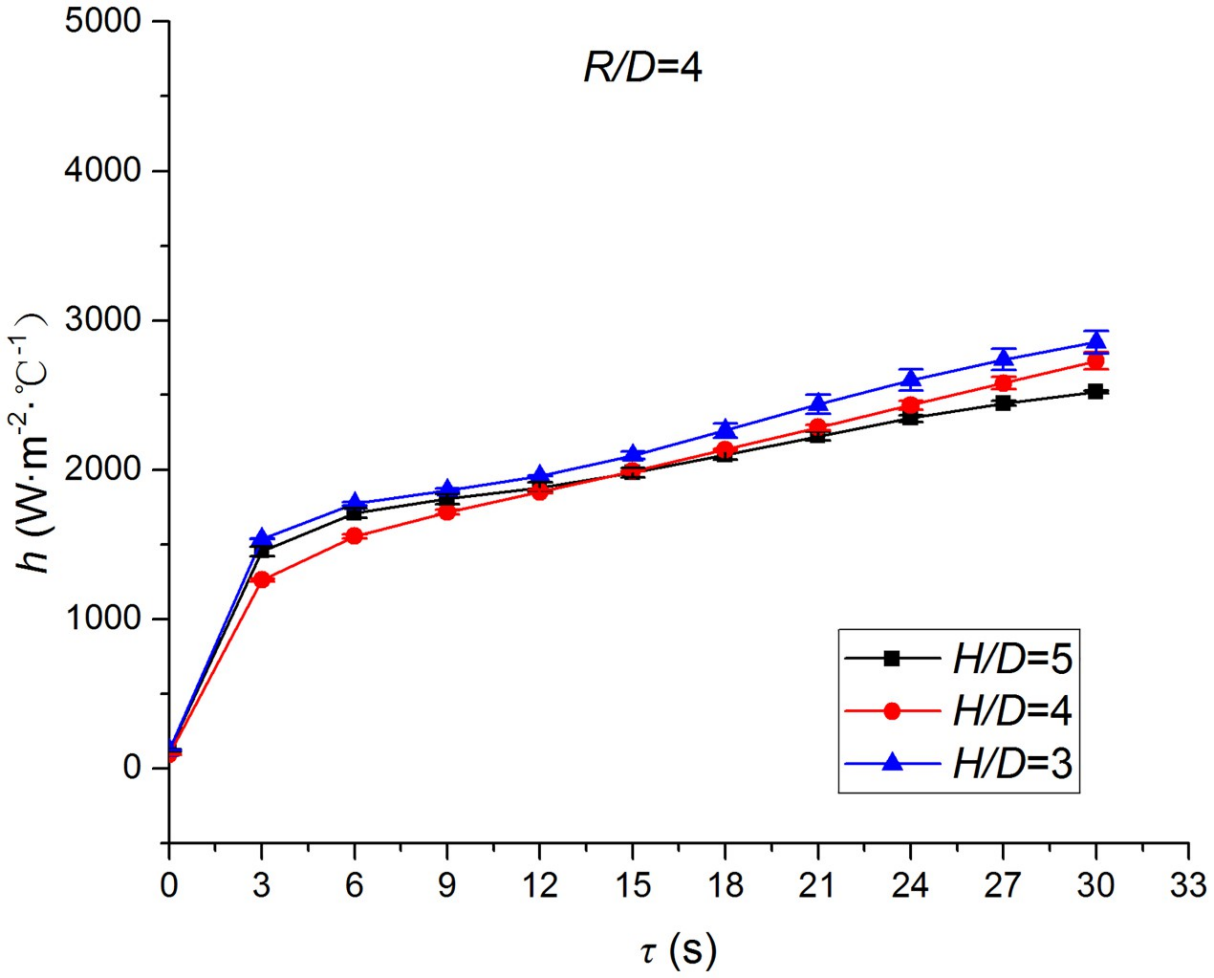
Convective heat transfer coefficient at *R/D* = 4.

**Fig 31:**
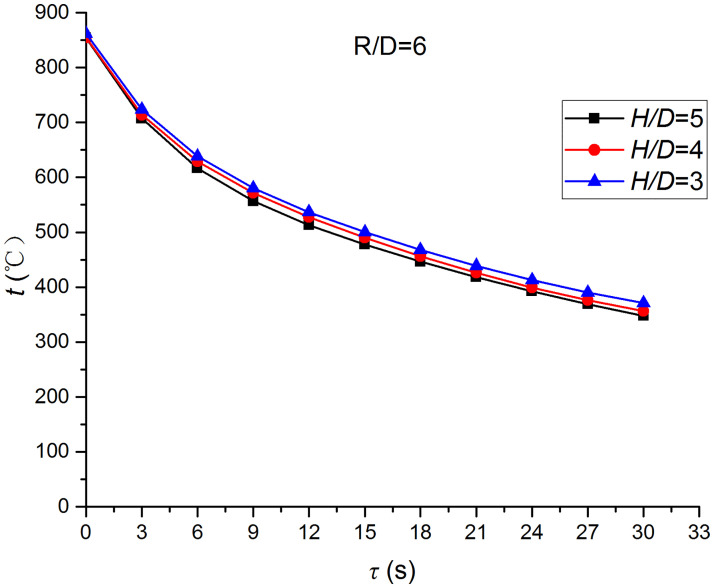
Surface temperature at *R/D* = 6.

**Fig 32:**
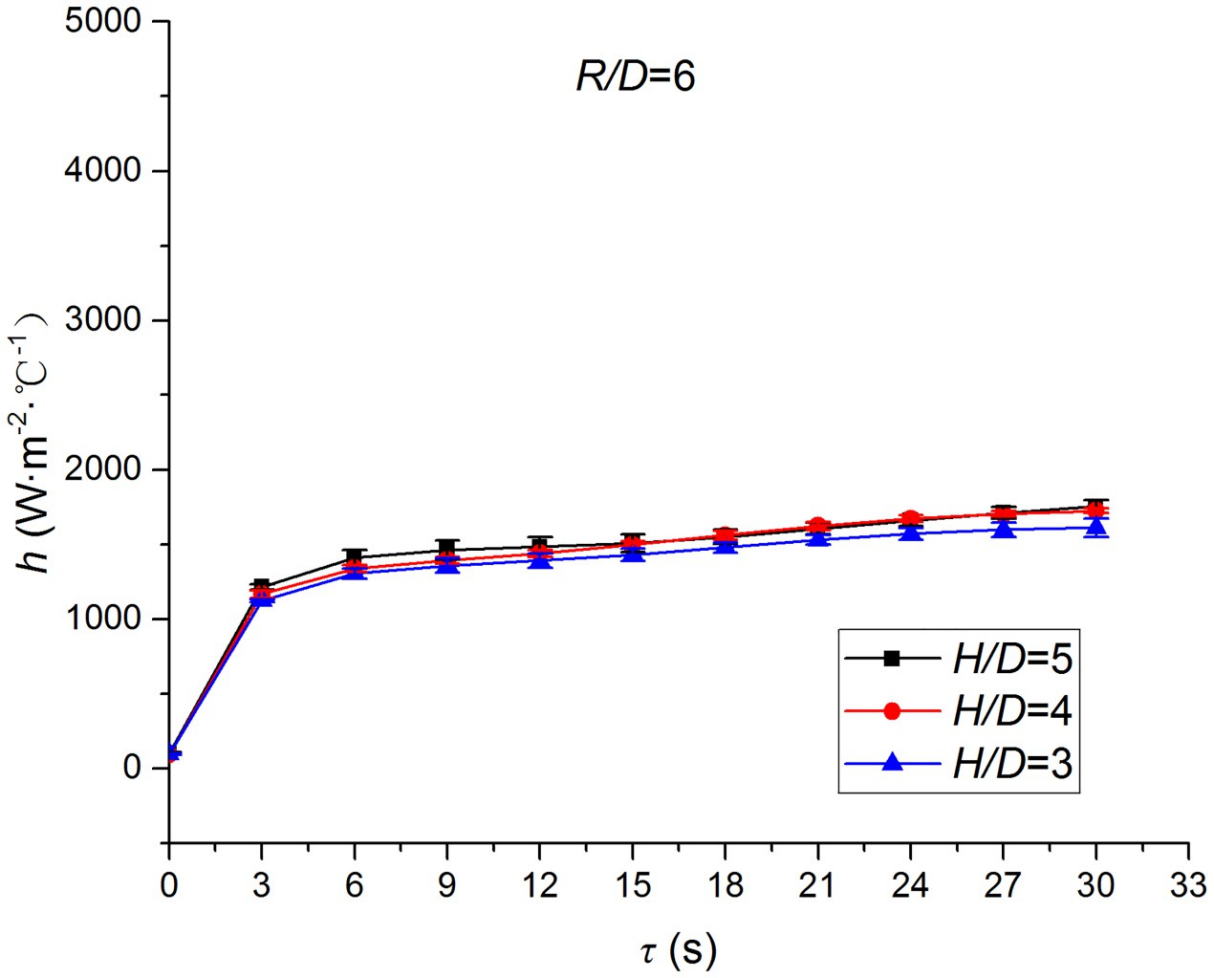
Convective heat transfer coefficient at *R/D* = 6.

**Table 3:** Average convective heat transfer coefficient and *AD* at different heat transfer positions and nozzle-to-target distances.

	*H/D* = 3	*H/D* = 4	*H/D* = 4	*AD*
*R/D* = 0	3186.29	3235.79	2797.22	13.77%
*R/D* = 2	2258.20	2397.13	2291.94	5.79%
*R/D* = 4	1871.12	1875.17	2021.99	7.46%
*R/D* = 6	1406.05	1382.88	1317.60	6.29%

## Conclusions

In summary, a discrimination-experiment method was developed to investigate the transient heat transfer characteristics of the air jet impingement. This method is especially suitable for high temperature differences or intense heat transfer conditions. The transient heat transfer characteristics of the supersonic air impingement on a high-temperature target (860°C) and the effects of the thermophysical parameters and nozzle-to-target distance are systematically analyzed.

The obtained results can be summarized as follows:

1. The discrimination-experiment method can be used to investigate the transient heat transfer characteristics of the air jet impingement through one-dimensional calculations without installing thermocouples in the solid domains. The proposed method is based on discretizing the solid domains into mutually adiabatic test cylinders.
2. The heat transfer capacity was greatly improved using the supersonic air jet and became comparable with the water jet at certain instances. The convective heat transfer coefficient rapidly increased at the beginning of the jet impingement; however, the increasing trend slowed down after 3 s. Moreover, the heat transfer was found to be most intense at the center (TP1) and gradually decreased from the center to the edges.
3. The dependence of the thermophysical parameters on temperature had a significant impact on the test results. For instance, the relative error between *h*_*con*,*all*_ and *h*_var *y*_ gradually increased and reached the maximum value of 49.6 at the end of the jet impingement, where the main error source was identified as the change in the thermal conductivity.
4. At *H/D* = 3, 4, and 5, the heat transfer at the center of the interface (*R/D* = 0) was clearly affected by the nozzle-to-target distance, which was found to be significantly weaker at *H/D* = 3. At the other locations (*R/D* = 2, 4, and 6), the convective heat transfer coefficient was not much influenced by the nozzle-to-target distance.

## Supporting information

S1 Table The coefficients of Eq (8) at H/D = 3, 4, and 5. (DOCX)

## Abbreviations

*c*_*p*_: specific heat capacity of test cylinder (J·kg^-1^·°C^-1^)
*D*: throat diameter of Laval nozzle (m)
*h*: convective heat transfer coefficient (W·m^-2^·°C^-1^)
*H*: nozzle-to-target distance (m)
*H/D*: non-dimensional nozzle-to-target distance ratio
*h*_*con*,*_: convective heat transfer coefficient when one or all thermo physical parameters are constant, i.e., *h*_*con*,*ρ*_, *h*_*con*,*λ*_, $h_{con,c_{p}}$, or *h*_*con*,*all*_ (W·m^-2^·°C^-1^)
*h*_*con*,var *y*_: convective heat transfer coefficient when all thermo physical parameters are varied with temperature (W·m^-2^·°C^-1^)
*h*_*dev*,*_: convective heat transfer coefficient when one of the experimental parameters is considered to pocess an artificial deviation according to its range of uncertainty, i.e., *h*_*dev*,*ρ*_, *h*_*dev*,*λ*_, $h_{dev,c_{p}}$, $h_{dev,t_{\infty }}$ and $h_{dev,t_{s}}$(W·m^-2^·°C^-1^)
*h* ^*i*^: convective heat transfer coefficient at time *i* (W·m^-2^·°C^-1^)
*I*: time number of the node
*L*: length of the test cylinder (m)
*N*: location number of the node
*Q*: heat flux (W·m^-2^)
*q* ^*i*^: heat flux at time *i* (W·m^-2^)
*R*: radius of the heat transfer position (m)
*R/D*: non-dimensional distance of test position
*T*: end time of impingement (s)
*t*_0_: initial temperature of test cylinder (°C)
*t*_∞_: temperature of air jet (°C)
*t*_*b*_: measured temperature of the painted surface when the emissivity is set at ε_b_
*t*_*m*_: measured temperature of the unpainted surface when the emissivity is set at 1
$t_{n}^{i}$: temperature of node *n* at time *i* (°C)
*t*_*s*_: temperature of impingement surface (°C)
*δt*: time step (s)
*δx*: space step (m)
*ε*_*b*_: emissivity of the black paint
*ε*_*s*_: emissivity of the unpainted surface
*λ*: thermal conductivity of the test cylinder (W·m^-1^·°C^-1^)
*ρ*: density of test cylinder (kg·m^-3^)
*σh*: probability distribution similar to the uncertainty probability distribution of all the primitive parameters (W·m^-2^·°C^-1^)
