# Supplementary material for: Investigation of the transient heat transfer to a supersonic air jet impinging on a high-temperature plate based on a discrimination-experiment method
Source: PLoS One. 2022 Mar 14;17(3):e0264968. doi: 10.1371/journal.pone.0264968 (PMC8920269; doi:10.1371/journal.pone.0264968)
Supplement: S1 Table — (DOCX) [file pone.0264968.s001.docx]

**Supporting Information: Table S1**

The coefficients of Eq. (8) at H/D=3, 4 and 5 are present in table 1, 2 and 3, respectively.

**Table 1．Coefficients of Eq. (8) at *H/D*=3.**

|  | *R/D*=0 | *R/D*=2 | *R/D*=4 | *R/D*=6 |
| --- | --- | --- | --- | --- |
| a_1_ | 8.116E-11 | 7.333E-11 | 1.732E-10 | 7.782E-11 |
| a_2_ | -2.742E-08 | -2.346E-08 | -5.486E-08 | -2.554E-08 |
| a_3_ | 3.921E-06 | 3.146E-06 | 7.230E-06 | 3.513E-06 |
| a_4_ | -3.092E-04 | -2.295E-04 | -5.131E-04 | -2.627E-04 |
| a_5_ | 0.0147 | 0.009934 | 0.02124 | 0.01161 |
| a_6_ | -0.4347 | -0.2655 | -0.5226 | -0.3103 |
| a_7_ | 8.096 | 4.697 | 7.703 | 5.093 |
| a_8_ | -97.86 | -66.94 | -78.4 | -59.08 |
| a_9_ | 859.2 | 859 | 858.4 | 861.7 |

**Table 2．Coefficients of Eq. (8) at H/D=4.**

|  | *R/D*=0 | *R/D*=2 | *R/D*=4 | *R/D*=6 |
| --- | --- | --- | --- | --- |
| a_1_ | 2.123E-10 | 9.775E-11 | 8.928E-11 | 1.181E-10 |
| a_2_ | -6.704E-08 | -3.073E-08 | -2.785E-08 | -3.696E-08 |
| a_3_ | 8.844E-06 | 4.032E-06 | 3.630E-06 | 4.823E-06 |
| a_4_ | -6.338E-04 | -2.870E-04 | -2.572E-04 | -3.409E-04 |
| a_5_ | 0.02692 | 0.01207 | 0.0108 | 0.01419 |
| a_6_ | -0.6977 | -0.309 | -0.278 | -0.356 |
| a_7_ | 11.15 | 4.993 | 4.594 | 5.463 |
| a_8_ | -113.8 | -62.55 | -60.45 | -59.84 |
| a_9_ | 853.8 | 856.3 | 855.6 | 854.5 |

**Table 3．Coefficients of Eq. (8) at H/D=5.**

|  | *R/D*=0 | *R/D*=2 | *R/D*=4 | *R/D*=6 |
| --- | --- | --- | --- | --- |
| a_1_ | 1.715E-10 | 8.668E-11 | 1.235E-10 | 6.042E-11 |
| a_2_ | -5.574E-08 | -2.744E-08 | -3.974E-08 | -2.048E-08 |
| a_3_ | 7.598E-06 | 3.609E-06 | 5.341E-06 | 2.929E-06 |
| a_4_ | -5.655E-04 | -2.561E-04 | -3.887E-04 | -2.296E-04 |
| a_5_ | 0.0251 | 0.01074 | 0.01663 | 0.01072 |
| a_6_ | -0.6838 | -0.2784 | -0.4275 | -0.3048 |
| a_7_ | 11.47 | 4.826 | 6.697 | 5.301 |
| a_8_ | -118.8 | -67.9 | -73.44 | -62.68 |
| a_9_ | 853.2 | 860.3 | 859.1 | 853.9 |
